# Natural Antioxidants, Tyrosinase and Acetylcholinesterase Inhibitors from *Cercis glabra* Leaves

**DOI:** 10.3390/molecules27248667

**Published:** 2022-12-07

**Authors:** Yueyue Lou, Ting Xu, Huaqiang Cao, Qiuyue Zhao, Pengpai Zhang, Penghua Shu

**Affiliations:** 1 School of Life Sciences, Henan University, Kaifeng 475004, China; 2 Engineering Research Center for Applied Microbiology of Henan Province, Kaifeng 475004, China; 3 Food and Pharmacy College, Xuchang University, 88 Bayi Road, Xuchang 461000, China

**Keywords:** *Cercis glabra*, flavonoids, antioxidant, tyrosinase, acetylcholinesterase

## Abstract

*Cercis glabra* is a plant belonging to the legume family, whose flowers and barks are commonly used as food and traditional Chinese medicines. However, its leaves are usually disposed of as wastes. This research comprehensively investigated the bioactive constituents of *C. glabra* leaves, and two new phenolic, ceroffesters A-B (**1**–**2**) and thirteen known compounds (**3**–**15**) were isolated. Their structures were elucidated by spectroscopic methods such as nuclear magnetic resonance (1D NMR and 2D NMR), high-resolution electrospray ionization mass spectra (HR-ESI-MS), optical rotatory dispersion (ORD) and electronic circular dichroism (ECD). All of them were assessed for their antioxidant activities through ABTS, DPPH and PTIO methodologies, and evaluated for inhibitory activities against two enzymes (mushroom tyrosinase and acetylcholinesterase). As a result, compounds **3**–**6**, **10** and **13** exhibited evident antioxidant activities. Meanwhile, compounds **5**, **10** and **13** showed the most potent tyrosinase inhibitory activities, with IC_50_ of 0.64, 0.65 and 0.59 mM, and compared with the positive control of 0.63 mM (kojic acid). In the initial concentration of 1 mg/mL, compounds **3**, **5** and **6** demonstrated moderate inhibitory activities against acetylcholinesterase with 85.27 ± 0.06%, 83.65 ± 0.48% and 82.21 ± 0.09%, respectively, compared with the positive control of 91.17 ± 0.23% (donepezil). These bioactive components could be promising antioxidants, tyrosinase and acetylcholinesterase inhibitors.

## 1. Introduction

Antioxidation, whitening and Alzheimer’s disease (AD) have always been the focus of attention in the fields of health and medicine. Antioxidants have been widely used as food additives to reduce or avoid the degradation of food and improve its palatability. In addition, antioxidants are important in preventing diseases [1]. The human body will produce a series of free radicals during normal metabolism. Reasonable concentrations of free radicals play an active role in human metabolism to maintain cell stability and transmit signals, while excessive free radicals will induce oxidative stress, causing cell aging and damage. Oxidative stress is associated with the pathogenesis of skin diseases, inflammation, atherosclerosis, neurological diseases and diabetes [2,3]. Therefore, inhibiting the production of numerous free radicals is of importance to prevent damage caused by oxidation. The antioxidant effect plays an active role in inhibiting the process of melanin synthesis, which could be produced in the organism with the catalysis of tyrosinase [4]. Tyrosinase plays a key role in skin, hair and eye coloration and in protecting skin from ultraviolet damage. However, excessive tyrosinase can cause uneven pigment distribution and localized pigmentation, which can lead to freckles, chloasma and even malignant melanoma [5]. Therefore, whitening by inhibiting tyrosinase activity has been developed as an important means to treat excessive melanin [6].

Acetylcholinesterase (AChE) promotes the degradation of acetylcholine at synapses and neuromuscular junctions, leading to the termination of nerve impulses. Decreased acetylcholine levels are one of the major causes of cognitive impairment in Alzheimer’s disease [7,8]. The inhibition of AChE is one of the key strategies for treating Alzheimer’s disease. Until now, the current existing AChE inhibitors have the disadvantages of unanticipated side effects and short validity periods, thus finding relatively safe and effective inhibitors has become a focus of research in the medical field [9].

Human health has become the focus of food development. Functional ingredients and functional food are investigated and have become a trend for food research worldwide [10]. Ingredients with extensive pharmacological activities can be found in many functional foods, including traditional Chinese medicine and edible plants [11].

*Cercis* is one genera of the legume family and contains about eight species, namely *C. glabra*, *C. chinensis*, *C. canadensi*, *C. siliquastrum*, *C. griffithii*, *C. chuniana*, *C. chingii* and *C. racemose*. These plants are widely grown in Western and Central Asia, Southern Europe and North America [12]. *Cercis* flowers are rich in volatile oil, amino acids [13], and have long been prepared as salads, snacks and scented teas by the population. The *Cercis* flowers’ pigment is often produced as a natural edible red pigment in the food industry [14]. In addition to its edible and nutritional value, *Cercis* plants also have a variety of medicinal values. The flowers, barks and fruits from *Cercis* plants have been used as traditional Chinese medicines to treat various diseases, such as wind-dampness, cold-arthralgia, carbuncle and swelling. Phytochemical studies of the *Cercis* genus have found the isolation of stilbenes, flavonoids, lignans, phenolic acids, cyanogenic glycosides and others [15], while pharmacological investigations have revealed anti-inflammatory, antioxidant, antithrombotic, bacteriostatic, anticoagulant and hypoglycemic activities [16,17,18]. However, compared with other genera of legumes, the previous studies on the chemical constituents and bioactivities of the *Cercis* plant are very scarce. More attention should be paid to it for the further development and utilization of *Cercis* resources.

*C. glabra* is a megaphanerophyte whose flowers are highly ornamental and are often planted in courtyards and roadsides [12]. In recent years, in addition to its ornamental value, research on its medicinal value has gradually progressed from the total plant extracts to the effective monomeric compounds obtained from plants [19]. In the process of searching for effective, novel and safe antioxidants as well as enzymes inhibitors from natural plants [20,21], the 95% ethanolic extract from *C. glabra* leaves has strong inhibitory activity. A further phytochemical investigation on the ethanolic extract led to two new esters of ceroffesters A-B (**1**–**2**), eight flavonoids (**3**–**10**) and five others (**11**–**15**) which were isolated and identified. Herein, details of the isolation, structure elucidation and biological activities of these isolated compounds are described.

## 2. Results and Discussion

### 2.1. Structure Elucidation of Compounds ***1**–**15***

**Figure 1:**
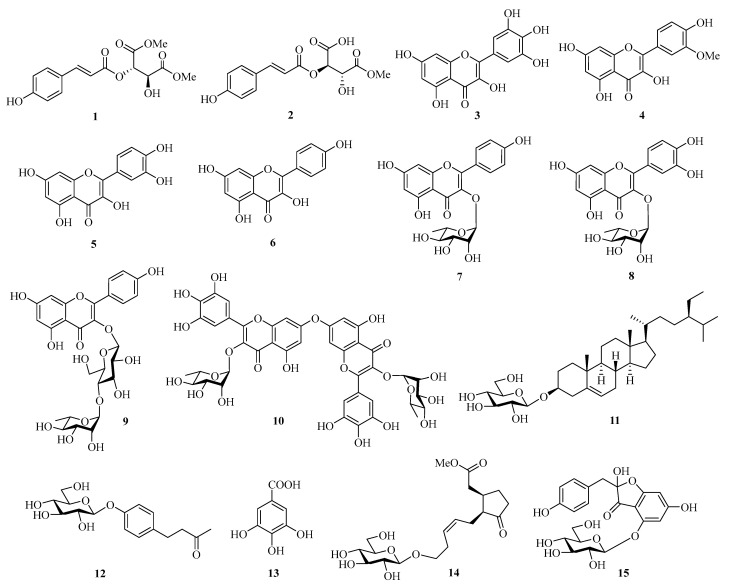
Structures of compounds **1**–**15**.

Two new compounds, ceroffesters A-B (**1**–**2**) and thirteen known ones (**3**–**15**) were isolated from the leaves of *C. glabra* (Figure 1).

By spectroscopic data analysis, they were determined as ceroffester A (**1**), ceroffester B (**2**), myricetin (**3**) [22], isorhamnetin (**4**) [23], quercetin (**5**) [24], kaempferol (**6**) [24], afzelin (**7**) [25], quercitrin (**8**) [26], kaempferol-3-*O*-rutinoside (**9**) [27], myricetin-3-*O*-rhamnoside-(C7-I-*O*-C7-II)-myricetin-3-*O*-rhamnoside (**10**) [28], carotene (**11**) [29], 4-(4′-Hydroxyphenyl)-2-butanone-4′-*O*-*β*-d-glucopyranoside (**12**) [30], gallic acid (**13**) [31], methyl-*β*-d-glucopyranosyl tuberonate (**14**) [32], hovetrichoside C (**15**) [33], by comparing their NMR data with those reported in the literature. Compounds **1**–**4**, **9**–**10** and **12**–**15** were reported from the *C. glabra* leaves for the first time, and compounds **1**–**2** and **10** were first reported from the *Cercis* leaves.

**Figure 2:**
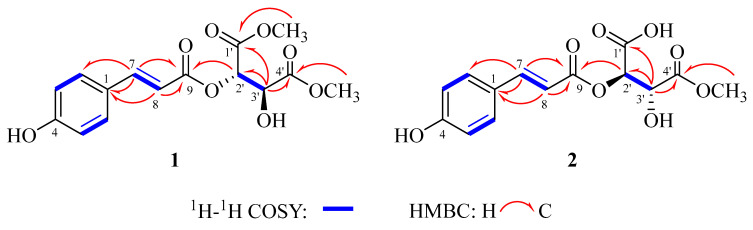
Key ^1^H-^1^H COSY and HMBC correlations for compounds **1** and **2**.

Compound **1** was obtained as a white powder. The IR absorption band at 3424 cm^−1^ suggested the presence of hydroxyl groups. The IR absorption bands at 1729, 1708 and 1695 cm^−1^ suggested the presence of three carbonyl groups (Figure S3). The molecular formula C_15_H_16_O_8_ was established based on its quasi-molecular ion peak at *m*/*z* 325.0932 [M + H]^+^ (calculated for C_15_H_17_O_8_, 325.0923) in the HR-ESI-MS spectrum (Figure S1), with eight degrees of unsaturation. The ^1^H-NMR spectrum (Figure S4) revealed the signals of an AA′BB′ system at δ_H_ 7.48 (2H, d, *J* = 8.0 Hz, H-2/H-6), 6.82 (2H, d, *J* = 8.0 Hz, H-3/H-5), a *trans*-double bond (*δ*_H_ 7.73 (1H, d, *J* = 16.0 Hz, H-7), 6.36 (1H, d, *J* = 16.0 Hz, H-8)), suggesting the presence of a *trans*-coumaroyl group in **1**. Additionally, the ^1^H-NMR spectrum displayed two doublets at *δ*_H_ 5.57 (1H, d, *J* = 2.4 Hz, H-2′) and 4.81 (1H, d, *J* = 2.4 Hz, H-3′), corresponding to protons of two oxygenated methines, and two methoxyl groups *δ*_H_ 3.79 (3H, s, 1′-OC*H*_3_) and 3.74 (3H, s, 4′-OC*H*_3_). In the ^13^C-NMR and DEPT spectra (Figures S5 and S6), aside from nine typical carbon signals assigned for the *trans*-coumaroyl group (*δ*_C_ 167.8 (C-9), 161.7 (C-4), 148.3 (C-7), 131.6 (C-2/C-6), 127.1 (C-1), 117.0 (C-3/C-5), 113.8 (C-8)), another six signals were observed at *δ*_C_ 172.4 (C-4′), 169.3 (C-1′), 74.8 (C-2′), 72.0 (C-3′), 53.3 (1′-O*C*H_3_) and 53.2 (4′-O*C*H_3_), which were attributed to a tartaric acid dimethyl ester. The ^1^H-^1^H COSY correlations (Figure S8) between H-2′ and H-3′, along with the HMBC correlations (Figure S9) from 1′-OC*H*_3_ to C-1′ and 4′-OC*H*_3_ to C-4′ also supported the presence of a tartaric acid dimethyl ester (Figure 2). The downfield chemical shifts of C-2′ and H-2′, together with the HMBC correlations between H-2′ and C-9, revealed that the *trans*-coumaroyl group was connected to the tartaric acid dimethyl ester at C-2′. Therefore, the structure of compound **1** was identified as *trans*-4-coumaroyltartaric acid dimethyl ester. The NMR data and planar structure of compound **1** were similar to those of a known compound isolated from the fruit of *Cornus officinalis*, namely (2′*R*, 3′*R*)-*trans*-4-coumaroyltartaric acid dimethyl ester (ceroffester D) [34]. The positive optical rotation of ceroffester D was determined as (2′*R*, 3′*R*), while compound **1** was negative optical rotation, and its absolute and relative stereochemistry was determined as 2′S and 3′S. Finally, the structure of compound **1** was identified as (2′*S*, 3′*S*)-*trans*-4-coumaroyltartaric acid dimethyl ester, namely ceroffester A.

**Figure 3:**
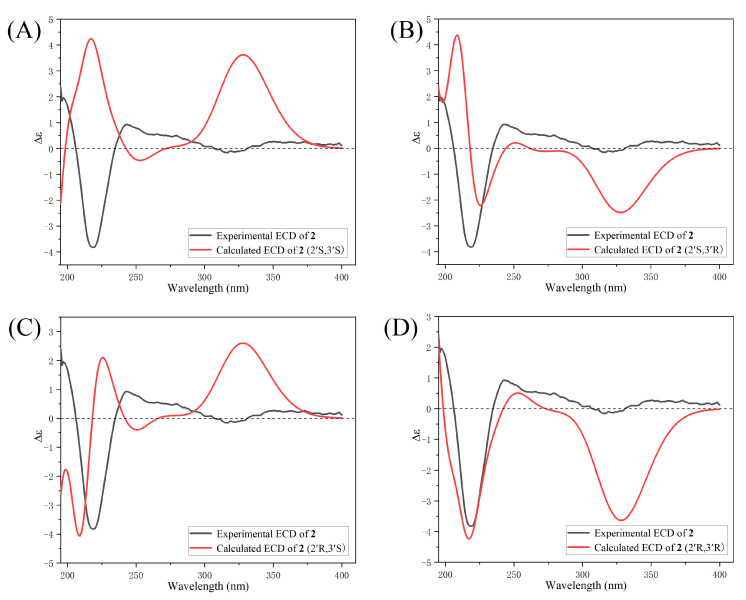
(**A**): Experimental ECD of **2** and calculated ECD of (2′S, 3′S) -**2**; (**B**): Experimental ECD of **2** and calculated ECD of (2′S, 3′R) -**2**; (**C**): Experimental ECD of **2** and calculated ECD of (2′R, 3′S) -**2**; (**D**): Experimental ECD of **2** and calculated ECD of (2′R, 3′R) -**2**.

Compound **2** was obtained as a white powder. The IR absorption bands at 3434, 1739 and 1698 cm^−1^ suggested the presence of hydroxyl groups and carbonyl groups (Figure S13). The molecular formula C_14_H_14_O_8_ was established based on its quasi-molecular ion peak at *m*/*z* 311.0765 [M + H]^+^ (calculated for C_14_H_15_O_8_, 311.0767) in the HR-ESI-MS spectrum (Figure S11), with eight degrees of unsaturation. The ^1^H-NMR and ^13^C-NMR spectra (Figures S14 and S15) of **2** were very similar to those of **1**, except that one methoxyl group was absent in **2** compared to **1**. The above speculations were confirmed by the key ^1^H-^1^H COSY (Figure S18) and HMBC correlations (Figure 2). The HMBC correlations (Figure S19) from 4′-OC*H*_3_ to C-4′ revealed that only one methoxyl group was attached to C-4′ (Figure 2). Therefore, the planar structure of **2** was identified as *trans*-4-coumaroyl-4′-methoxyl-tartaric acid. In order to determine the absolute configuration of **2**, the ECD spectra for (2′*R*, 3′*R*)-**2** and its three isomers, (2′*S*, 3′*S*)-**2**, (2′*R*, 3′*S*)-**2** and (2′*S*, 3′*R*)-**2**, were calculated by the time-dependent density functional theory (TD-DFT) calculations (Figure 3). The measured ECD spectrum of **2** fits well with the calculated ECD of the (2′*R*, 3′*R*)-**2**, and is opposite to that of its enantiomer (2′*S*, 3′*S*)-**2** (Figure 3A,D). Therefore, Compound **2** was identified as (2′*R*, 3′*R*)-*trans*-4-coumaroyl-4′-methoxyl-tartaric acid, namely ceroffester B.

Myricetin (**3**). Yellow powder. ^1^H-NMR (CD_3_OD, 400 MHz) δ: 7.76 (2H, d, *J* = 8.8 Hz, H-2′/6′), 6.38 (1H, d, *J* = 2.0 Hz, H-8), 6.18 (1H, d, *J* = 2.0 Hz, H-6); ^13^C-NMR (CD_3_OD, 100 MHz) δ: 177.4 (C-4), 165.7 (C-7), 162.6 (C-5), 158.3 (C-9), 148.1 (C-2), 146.9 (C-3′/5′), 137.5 (C-3), 137.1 (C-4′), 123.2 (C-1′), 108.7 (C-2′/6′), 104.3 (C-10), 99.5 (C-6), 94.5 (C-8) [22].

Isorhamnetin (**4**). Yellow powder. ^1^H-NMR (DMSO-d_6_, 400 MHz) δ: 12.47 (1H, s, 5-OH), 10.81 (1H, s, 7-OH), 9.77 (1H, s, 4′-OH), 9.45 (1H, s, 3-OH), 7.75 (1H, d, *J* = 2.0 Hz, H-2′), 7.69 (1H, dd, *J* = 8.4, 2.0 Hz, H-6′), 6.94 (1H, d, *J* = 8.8 Hz, H-5′), 6.48 (1H, d, *J* = 2.0 Hz, H-8), 6.19 (1H, d, *J* = 2.0 Hz, H-6), 3.84 (3H, s, 3′-OCH_3_); ^13^C-NMR (DMSO-d_6_, 100 MHz) δ: 176.8 (C-4), 164.8 (C-7), 161.6 (C-5), 157.1 (C-9), 149.7 (C-3′), 148.3 (C-4′), 147.5 (C-2), 136.7 (C-3), 122.9 (C-1′), 122.6 (C-6′), 116.5 (C-5′), 112.7 (C-2′), 103.9 (C-10), 99.1 (C-6), 94.5 (C-8), 56.7 (3′-OCH_3_) [23].

Quercetin (**5**). Yellow powder. ^1^H-NMR (CD_3_OD, 400 MHz) δ: 7.74 (1H, d, *J* = 2.0 Hz, H-2′), 7.63 (1H, dd, *J* = 8.4, 2.0 Hz, H-6′), 6.88 (1H, d, *J* = 8.8 Hz, H-5′), 6.39 (1H, d, *J* = 1.6 Hz, H-8), 6.18 (1H, d, *J* = 1.6 Hz, H-6); ^13^C-NMR (CD_3_OD, 100 MHz) δ: 177.5 (C-4), 165.7 (C-7), 162.6 (C-5), 158.4 (C-9), 148.9 (C-4′), 148.2 (C-2), 146.4 (C-3′), 137.4 (C-3), 124.3 (C-1′), 121.8 (C-6′), 116.4 (C-5′), 116.2 (C-2′), 104.7 (C-10), 99.4 (C-6), 94.6 (C-8) [24].

Kaempferol (**6**). Yellow crystal. ^1^H-NMR (CD_3_COCD_3_, 400 MHz) δ: 8.14 (2H, d, *J* = 8.8 Hz, H-2′/6′), 7.00 (2H, d, *J* = 8.8 Hz, H-3′/5′), 6.52 (1H, d, *J* = 2.0 Hz, H-8), 6.23 (1H, d, *J* = 2.0 Hz, H-6); ^13^C-NMR (CD_3_COCD_3_, 100 MHz) δ: 177.6 (C-4), 166.0 (C-7), 163.3 (C-5), 161.2 (C-4′), 158.8 (C-9), 148.1 (C-2), 137.7 (C-3), 131.5 (C-2′/6′), 124.4 (C-1′), 117.4 (C-3′/5′), 105.2 (C-10), 100.2 (C-6), 95.6 (C-8) [24].

Afzelin (**7**). Yellow powder. ^1^H-NMR (CD_3_OD, 400 MHz) δ: 7.65 (2H, d, *J* = 8.8 Hz, H-2′/6′), 6.93 (2H, d, *J* = 8.8 Hz, H-3′/5′), 6.37 (1H, d, *J* = 2.4 Hz, H-8), 6.19 (1H, d, *J* = 2.4 Hz, H-6), 5.38 (1H, d, *J* = 1.6 Hz, H-1′′), 4.23–3.32 (4H, m, H-2′′/3′′/5′′/4′′), 0.92 (3H, d, *J* = 6.0 Hz, H-6′′); ^13^C-NMR (CD_3_OD, 100 MHz) δ: 179.8 (C-4), 166.0 (C-7), 163.4 (C-4′), 161.7 (C-5), 159.4 (C-9), 158.7 (C-2), 136.4 (C-3), 132.1 (C-2′/6′), 122.8 (C-1′), 116.7 (C-3′/5′), 106.1 (C-10), 103.7 (C-1′′), 100.0 (C-6), 94.9 (C-8), 73.4 (C-4′′), 72.3 (C-3′′), 72.2 (C-2′′), 72.1 (C-5′′), 17.8 (C-6′′) [25].

Quercitrin (**8**). Yellow powder. ^1^H-NMR (CD_3_OD, 400 MHz) δ: 7.34 (1H, d, *J* = 2.0 Hz, H-2′), 7.31 (1H, dd, *J* = 8.0, 2.0 Hz, H-6′), 6.91 (1H, d, *J* = 8.4 Hz, H-5′), 6.37 (1H, d, *J* =2.4 Hz, H-8), 6.20 (1H, d, *J* = 2.0 Hz, H-6), 5.35 (1H, d, *J* = 1.6 Hz, H-1′′), 4.22 (1H, dd, *J* = 3.6, 2.0 Hz, H-2′′), 3.75 (1H, dd, *J* = 9.2, 3.2 Hz, H-3′′), 3.42 (1H, m, H-5′′), 3.35 (1H, m, H-4′′), 0.94 ((3H, d, *J* = 6.0 Hz, H-6′′); ^13^C-NMR (CD_3_OD, 100 MHz) δ: 179.8 (C-4), 166.0(C-7), 163.4 (C-5), 159.5 (C-9), 158.7 (C-2), 149.9 (C-4′), 146.6 (C-3′), 136.4 (C-3), 123.1 (C-1′), 123.0 (C-6′), 117.1 (C-5′), 116.5 (C-2′), 106.1 (C-10), 103.7 (C-1′′), 100.0 (C-6), 94.9 (C-8), 73.4 (C-4′′), 72.3 (C-3′′), 72.2 (C-2′′), 72.1 (C-5′′), 17.8 (C-6′′) [26].

Kaempferol-3-*O*-rutinoside (**9**). Yellow powder. ^1^H-NMR (CD_3_OD, 400 MHz) δ: 8.06 (2H, d, *J* = 8.8 Hz, H-2′/6′), 6.88 (2H, d, *J* = 8.4 Hz, H-3′/5′), 6.36 (1H, s, H-8), 6.18 (1H, s, H-6), 5.10 (1H, d, *J* = 6.8 Hz, H-1′′), 4.52 (1H, s, H-1′′′), 3.81 (1H, d, *J* = 9.6 Hz, H-6′′), 3.64 (1H, d, *J* = 1.5 Hz, H-2′′′), 1.13 (3H, d, *J* = 6.4 Hz, H-6′′′); ^13^C-NMR (CD_3_OD, 100 MHz) δ: 179.3 (C-4), 167.6 (C-7), 163.0 (C-5), 161.6 (C-4′), 159.4 (C-9), 158.8 (C-2), 135.6 (C-3), 132.5 (C-2′/6′), 122.9 (C-1′), 116.3 (C-3′/5′), 105.4 (C-10), 104.9 (C-1′′), 102.6 (C-1′′′), 100.6 (C-6), 95.4 (C-8), 78.3 (C-3′′), 77.3 (C-5′′), 75.9 (C-2′′), 74.1 (C-4′′′), 72.4 (C-3′′′), 72.2 (C-2′′′), 71.6 (C-4′′), 69.9 (C-5′′′), 68.7 (C-6′′), 18.1 (C-6′′′) [27].

Myricetin-3-*O*-rhamnoside-(C7-I-*O*-C7-II)-myricetin-3-*O*-rhamnoside (**10**). Yellow powder. ^1^H-NMR (CD_3_OD, 400 MHz) δ: 6.92 (4H, s, H-2′/6′, I/II), 6.35 (2H, d, *J* = 2.0 Hz, H-8, I/II), 6.16 (2H, d, *J* = 2.0 Hz, H-6, I/II), 5.29 (1H, brs, H-1′′, I), 5.28 (1H, brs, H-1′′, II), 4.20 (2H, m, H-2′′, I/II), 3.77 (2H, m, H-3′′, I/II), 3.49 (2H, m, H-5′′, I/II), 3.28 (2H, m, H-4′′, I/II), 0.94 ((3H, d, *J* = 6.0 Hz, H-6′′, I), 0.92 ((3H, d, *J* = 6.0 Hz, H-6′′, II); ^13^C-NMR (CD_3_OD, 100 MHz) δ: 179.8 (C-4, I/II), 165.9 (C-7, I/II), 163.3 (C-5, I/II), 159.5 (C-2, I/II), 158.6 (C-9, I/II), 146.9 (C-3′/5′, I/II), 138.0 (C-4′, I/II), 136.4 (C-3, I/II), 122.0 (C-1′, I/II), 109.7 (C-2′/6′, I/II), 106.0 (C-10, I/II), 103.7 (C-1′′, I/II), 99.9 (C-6, I/II), 94.8 (C-8, I/II), 73.4 (C-4′′, I/II), 72.2 (C-3′′/5′′, I/II), 72.0 (C-2′′, I/II), 17.8 (C-6′′, I/II) [28].

Carotene (**11**). White powder. ^1^H-NMR (DMSO-*d_6_*, 400 MHz) δ: 5.32 (1H, d, *J* = 4.8 Hz, H-6), 4.43 (1H, t, *J* = 6.0 Hz, H-3), 4.22 (1H, d, *J* = 7.6 Hz, H-1′), 3.56 (1H, m, H-4′, 3.34 (1H, dd, *J* = 10.6, 5.4 Hz, H-6′), 3.05 (2H, q, *J* = 5.2 Hz, H-3′/2′), 2.91 (1H, m, H-5′), 0.96 (3H, s, H-19), 0.90 (3H, d, *J* = 6.4 Hz, H-21), 0.85–0.79 (9H, s, H-29/27/26), 0.65 (3H, s, H-18); ^13^C-NMR (DMSO-*d_6_*, 100 MHz) δ: 141.4 (C-5), 122.1 (C-6), 101.8 (C-1′), 77.9 (C-3′), 77.7 (C-5′), 77.7 (C-3), 74.4 (C-2′), 71.0 (C-4′), 62.0 (C-6′), 57.1 (C-17), 56.4 (C-14), 50.4 (C-9), 46.1 (C-24), 42.8 (C-13), 40.2 (C-12), 39.3 (C-4), 37.8 (C-1), 37.1 (C-10), 36.4 (C-20), 34.3 (C-22), 32.4 (C-8), 32.3 (C-7), 30.2 (C-2), 29.6 (C-25), 28.7 (C-16), 26.4 (C-23), 24.8 (C-15), 23.5 (C-28), 21.5 (C-11), 20.6 (C-26), 20.0 (C-19), 19.9 (C-27), 19.5 (C-21), 12.7 (C-29), 12.6 (C-18) [29].

4-(4′-Hydroxyphenyl)-2-butanone-4′-*O*-β-d-glucopyranoside (**12**). Colorless syrup. ^1^H-NMR (CD_3_OD, 400 MHz) δ: 7.12 (2H, d, *J* = 8.4 Hz, H-7/9), 7.01 (2H, d, *J* = 8.4 Hz, H-6/10), 4.86 (1H, d, *J* = 8.0 Hz, H-1′), 3.31–3.90 (6H, m, H-2′/3′/4′/5′/6a′/6b′), 2.78 (4H, m, H-3/4), 2.11 (3H, s, H-1); ^13^C-NMR (CD_3_OD, 100 MHz) δ: 211.2 (C-2), 157.6 (C-8), 136.5 (C-5), 130.4 (C-6/10), 117.9 (C-7/9), 102.6 (C-1′), 78.2 (C-3′), 78.1 (C-5′), 75.0 (C-2′), 71.5 (C-4′), 62.6 (C-6′), 46.1 (C-4), 30.1 (C-1/3) [30].

Gallic acid (**13**). White crystal. ^1^H-NMR (CD_3_OD, 400 MHz) δ: 7.10 (2H, s, H-2/6); ^13^C-NMR (CD_3_OD, 100 MHz) δ: 170.9 (C-7), 146.3 (C-3/5), 139.5 (C-4), 122.2 (C-1), 110.5 (C-2/6) [31].

Methyl-β-d-glucopyranosyl tuberonate (**14**). colorless oil. ^1^H-NMR (CDCl_3_, 400 MHz). δ: 5.49 (1H, m, H-3′), 5.29 (1H, m, H-2′), 4.36 (1H, d, *J* = 7.4 Hz, Glc H-1), 3.87 (2H, m, H-5′b/Glc H-6b), 3.72 (3H, s, 1′′-OCH_3_), 3.57–3.28 (6H, m, H-5′a/Glc H-2/5/4/3/6a), 2.72 (1H, m, H-2′′b), 2.41 (5H, m, H-2′′a/1′/4′), 1.29–2.26 (6H, m, H-1/4b/5b/4a/2/5a); ^13^C-NMR (CDCl_3_, 100 MHz) δ: 219.6 (C-3), 172.9 (C-1′′), 128.1 (C-3′), 127.9 (C-2′), 103.0 (Glc C-1), 76.6 (Glc C-3), 75.8 (Glc C-5), 73.6 (Glc C-2), 69.8 (Glc C-4), 69.5 (C-5′), 61.8 (Glc C-6), 54.1 (C-2), 52.0 (1′′-OCH_3_), 38.9 (C-4), 38.0 (C-1/2′′), 28.1 (C-4′), 27.4 (C-5), 25.6 (C-1′) [32].

Hovetrichoside C (**15**). colorless syrup. ^1^H-NMR (CD_3_OD, 400 MHz) δ: 6.98 (2H, d, *J* = 8.4 Hz, H-2′/6′), 6.56 (2H, d, *J* = 8.4 Hz, H-3′/5′), 6.04 (1H, brs, H-5), 5.94 (1H, brs, H-7), 4.87 (1H, d, *J* = 6.0 Hz, H-1′′), 3.86–3.38 (6H, m, H-2′′/3′′/4′′/5′′/6′′), 3.08 (2H, brs, H-10); ^13^C-NMR (CD_3_OD, 100 MHz) δ: 197.1/197.0 (C-3), 174.7 (2C-8), 171.6 (2C-6), 158.6/158.4 (C-4), 157.4 (2C-4′), 132.7 (2C-2′/6′), 125.7 (2C-1′), 115.9 (2C-3′/5′), 107.8/107.7 (C-2), 103.8/103.6 (C-9), 101.8 (2C-1′′), 97.7/97.4 (C-5), 93.4/93.3 (C-7), 78.5 (2C-5′′), 77.5/77.4 (C-3′′), 74.2/74.1 (C-2′′), 71.3 (2C-4′′), 62.5 (2C-6′′), 42.3/42.1 (C-10) [33].

### 2.2. Antioxidant, Tyrosinase and Acetylcholinesterase Inhibitory Activities

**Figure 4:**
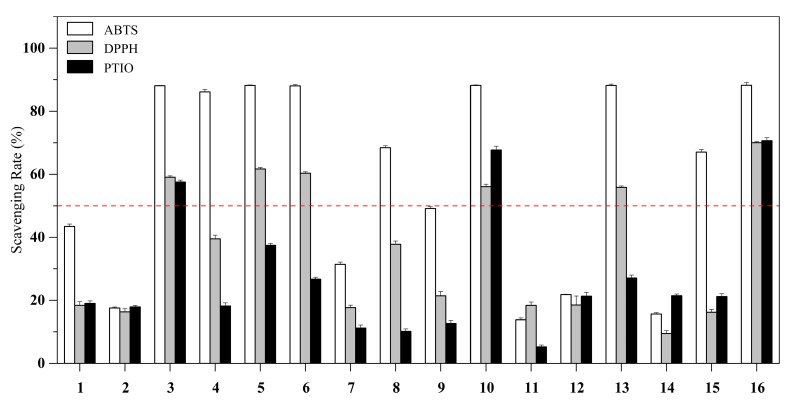
ABTS, DPPH and PTIO radical scavenging activities of compounds **1–15** and **16** (l-ascorbic acid), at initial concentration of 1 mg/mL.

The ABTS, DPPH and PTIO radicals have been widely used to evaluate the antioxidant capacity of natural products or extracts. In this study, fifteen isolates (at initial concentration of 1 mg/mL) from *C. glabra* leaves were explored with l-ascorbic acid as the positive control (Table 1 and Figure 4).

Six compounds showed ABTS radical scavenging rates of >86% at 1 mg/mL, including **3**–**6**, **10** and **13**. In particular, compounds **3** (myricetin), **5** (quercetin), **6** (kaempferol) and **10** (myricetin-3-*O*-rhamnoside-(C7-I-*O*-C7-II)-myricetin-3-*O*-rhamnoside) displayed higher IC_50_ values than l-ascorbic acid. Five compounds (**3**, **5**, **6**, **10** and **13**) showed similar IC_50_ values to l-ascorbic acid for DPPH radical scavenging assay. Moreover, three compounds (**3**, **5** and **10**) showed similar IC_50_ values to l-ascorbic acid in PTIO radical scavenging assay.

Of these bioactive components, all except gallic acid (**13**) are flavonoids. Therefore, the phenolic hydroxyl groups may be important for the radical scavenging activity. The ABTS, DPPH and PTIO methods are commonly used to evaluate the scavenging ability of free radicals. Thus, their reaction may be regarded as a direct antioxidant process. However, their scavenging mechanisms are in fact rather different. For example, DPPH and ABTS are nitrogen-centered radicals, while PTIO is an oxygen-centered radical. The ABTS radical is scavenged mainly involving one-electron transfer (ET), while DPPH and PTIO scavenging have been demonstrated to be involved in H^+^ transfer (HAT) [35]. As can been see from Table 1, compounds **3**, **5**, **6** and **10** could effectively scavenge three types of free radicals through ET and HAT pathways, indicating that they could be used as novel effective antioxidants.

In Table 2, most of the isolated compounds showed moderate-to-strong inhibitory activities against mushroom tyrosinase at 1 mg/mL. In particular, the new compounds **1**–**2** showed moderate tyrosinase inhibitory activities, while compounds **5**, **10** and **13** showed the most potent tyrosinase inhibitory activities, with IC_50_ of 0.64, 0.65 and 0.59 mM, respectively, while the positive control was 0.63 mM (kojic acid). Tyrosinase inhibitors, characterized by reducing melanin production and improving skin elasticity, are widely used in pharmaceutical and skincare industries [36]. However, current tyrosinase inhibitors may induce unwanted adverse reactions such as unequal pigmentation, skin irritation and even cancer [37]. Therefore, it is still necessary to explore safe, stable and effective tyrosinase inhibitors. As natural compounds extracted from medicinal plants, compounds **5**, **10** and **13** are of great pharmaceutical value both in cosmetics and pharmaceuticals, due to their potent biological properties.

The initial concentration was 1 mg/mL, and compounds **3**, **5** and **6** exhibited moderate acetylcholinesterase inhibitory activities, with a percentage inhibition value of 85.27 ± 0.06%, 83.65 ± 0.48% and 82.21 ± 0.09%, respectively, with donepezil used as the positive control (91.17 ± 0.23%). The inhibition of AChE serves as a strategy for the treatment of neurological disorders, including myasthenia gravis, glaucoma, Parkinson’s disease, senile dementia and ataxia [38]. The findings of this study reveal that ethanol extract from the *C. glabra* leaves may be a potential therapeutic agent for the treatment of Alzheimer’s disease, due to its phytochemical components.

Myricetin (**3**) and kaempferol (**6**) are two flavonoids widely distributed in natural plants [39,40] and have been previously reported to possess strong antioxidant and acetylcholinesterase inhibitory abilities [41,42,43,44]. Therefore, the results for **3** and **6** were generally consistent with the previous research. Quercetin (**5**) spread widely in fruits and vegetables [45], and was found to show antioxidant, tyrosinase and acetylcholinesterase inhibitory abilities [46,47], which fits well with the results of this research. Compound **10** has been tested to be a potential natural antioxidant [28]; however, the present study demonstrated for the first time that it is a promising tyrosinase inhibitor. Compound **13** was found to show antioxidant and tyrosinase inhibition activities [48,49], which was further confirmed.

## 3. Materials and Methods

### 3.1. General Experimental Procedures

Silica gel (100–200 or 300–400 meshes, Qingdao Marine Chemical Inc., Qingdao) was used for column chromatography, along with Sephadex LH-20 (25–100 µm, GE Healthcare Bioscience, Trenton, NJ, USA), ODS (50 μm, YMC, Shanghai, China) and Polyamide (200–400 mesh, Macklin, Shanghai, China). TLC was conducted with silica gel 60 F_254_ plates (0.20 mm Yantai Chemical Industry, Yantai, China), and the spots were detected by UV illumination (365 and 254 nm) and by spraying 10% H_2_SO_4_ followed by heating. UV, FT-IR and NMR spectra were recorded on Puxi TU-1950, FTIR-650 and Bruker AM-400 (400 MHz for ^1^H, 100 MHz for ^13^C) instruments, respectively. HR-ESI-MS dates were obtained from a Bruker micrOTOF II spectrometer. The optical rotatory dispersion (ORD) was obtained on JASCO P-2200 polarimeter and ECD spectra were obtained on a JASCO J-810 spectrometer. The absorbance was gained from Multiskan FC microplate reader (Thermo Fisher Scientific, Inc., Waltham, MA, USA).

### 3.2. Plant Materials

The fresh leaves of *C. glabra* were collected from Yanling Zhonglin Garden Engineering Co., Ltd., Xuchang, China, in May 2021. The species was identified by Prof. Lin Yang at Lanzhou Technology University. A specimen (No. SPH2021B) was stored in Xuchang University, China.

### 3.3. Extraction, Partition and Purification

**Scheme 1:**
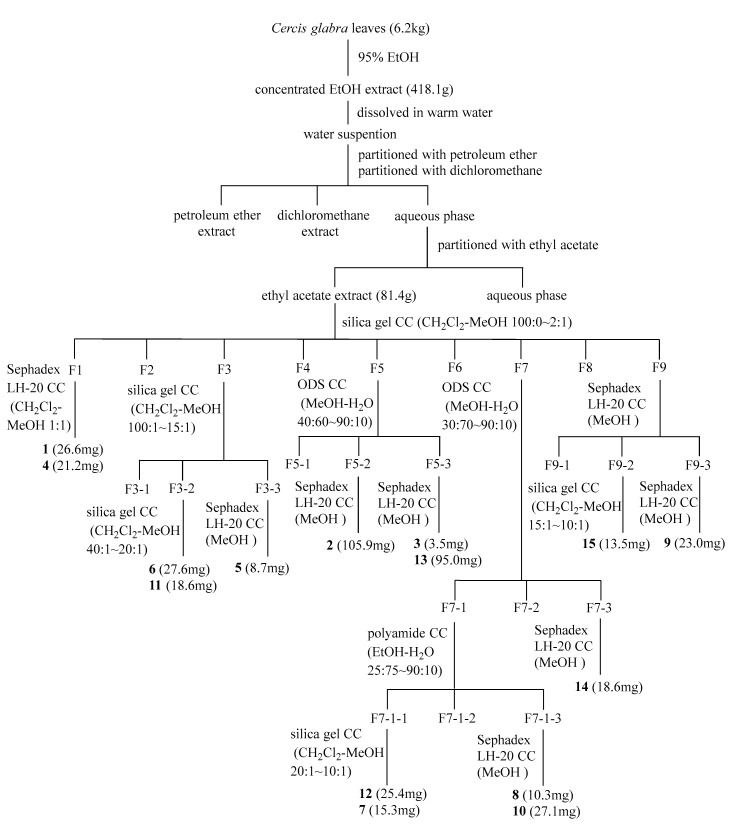
Extraction, fractionation and purification procedures of chemical constituents from *C. glabra* leaves.

According to Scheme 1, the dried *C. glabra* leaves (6.2 kg) were crushed and extracted three times with 50 L EtOH (95%) and soaked for 3 days at room temperature. The concentrated EtOH crude extract (418.1 g) was dissolved in warm H_2_O and partitioned with small polar solvent (petroleum ether, dichloromethane) and ethyl acetate (EtOAc). The EtOAc soluble fraction (81.4 g) was separated by chromatography on silica gel CC eluted with CH_2_Cl_2_-MeOH (100:0~2:1 *v*/*v*) to obtain nine fractions (F1–F9). The fraction F1 was separated by Sephadex LH-20 CC (CH_2_Cl_2_-MeOH 1:1 *v*/*v*) to yield compounds **1** (26.6 mg) and **4** (21.2 mg). The fraction F3 was fractionated on silica gel CC divided into multiple subfractions (F3-1–F3-3) by gradient elution with CH_2_Cl_2_-MeOH (100:1~15:1 *v*/*v*). Compounds **6** (27.6 mg) and **11** (18.6 mg) were purified from subfraction F3-2 on a silica gel CC eluted with gradient of CH_2_Cl_2_-MeOH (40:1~20:1 *v*/*v*). Subfraction F3-3 was chromatographed on Sephadex LH-20 CC eluted with MeOH to generate compound **5** (8.7 mg). Three subfractions (F5-1–F5-3) were obtained by ODS CC eluted with MeOH-H_2_O (40:60~90:10 *v*/*v*) from F5. Further purification gave compounds **2** (105.9 mg), **3** (3.5 mg) and **13** (95.0 mg) from subfractions F5-2 and F5-3. The fraction F7 was segmented into multiple subfractions (F7-1–F7-3) by ODS CC eluted with MeOH-H_2_O (30:70~90:10 *v*/*v*). Compounds **12** (25.4 mg), **7** (15.3 mg), **8** (10.3 mg) and **10** (27.1 mg) were obtained by the further separation of subfraction F7-1 utilizing Polyamide CC, Sephadex LH-20 CC and silica gel CC. Similarly, compound **14** (18.6 mg) was gained from subfraction F7-3. Fraction F9 was further isolated into compounds **15** (13.5 mg) and **9** (23.0 mg), which were applied to Sephadex LH-20 CC and silica gel CC.

**Ceroffester A** (**1**). White powder. ${\left[\alpha \right]}_{D}^{20}-$7.2 (c 0.6, MeOH). IR (KBr) *ν*_max_ 3424, 2962, 1729, 1708, 1695, 1635, 1608, 1513, 1442, 1355, 1309, 1284, 1253, 1224, 1164, 1068, 981, 835 cm^–1^. UV *λ*_max_ (MeOH) nm (log *ε*): 315 (5.07), 228 (4.82) (Figure S2). ^1^H-NMR and ^13^C-NMR data, in Table 3. HR-ESI-MS *m*/*z* 325.0932 [M + H]^+^ (calculated for C_15_H_17_O_8_, 325.0923).

**Ceroffester B** (**2**). White powder. ${\left[\alpha \right]}_{D}^{20}-$10.7 (c 1.2, MeOH). IR (KBr) *ν*_max_ 3434, 2960, 1739, 1698, 1625, 1606, 1515, 1434, 1349, 1315, 1286, 1243, 1224, 1199, 1157, 1074, 981, 825 cm^–1^. UV *λ*_max_ (MeOH) nm (log *ε*): 313 (5.17), 228 (4.89) (Figure S12). ^1^H-NMR and ^13^C-NMR data, in Table 3. HR-ESI-MS *m*/*z* 311.0765 [M + H]^+^ (calculated for C_14_H_15_O_8_, 311.0767).

Experimental data of compounds **3**–**15** can be found in Section 2.1.

### 3.4. Computational Section

Monte Carlo conformational searches were carried out by means of the Spartan’s 14 software using Merck Molecular Force Field (MMFF). The conformers with Boltzmann population of over 5% were chosen for ECD calculations, and then the conformers were initially optimized at B3LYP/6–31g level in gas. The theoretical calculation of ECD was conducted in MeOH using the time-dependent density functional theory (TD-DFT) at the B3LYP/6–31+g (d, p) level for all conformers of compound **2** and its isomers. Rotatory strengths for a total of 30 excited states were calculated. ECD spectra were generated using the program SpecDis 1.6 (University of Würzburg, Würzburg, Germany) and GraphPad Prism 5 (University of California, San Diego, CA, USA) from dipole-length rotational strengths by applying Gaussian band shapes with sigma = 0.3 eV.

### 3.5. ABTS Radical Scavenging Activity

The ABTS radical scavenging assay was modified according to the method with slight modifications [50]. Briefly, the ABTS radical cation was obtained by mixing ABTS diammonium salt stock solution (7.4 mM) with potassium persulfate (2.6 mM) in equal proportion and reacting it at 37 °C in darkness for 12–16 h. Before used, the absorbance of light green ABTS radical test solution at 745 nm was controlled to be 0.70 ± 0.02 by diluting with methanol [51]. Sample solution and ABTS methanol solution (10 µL:190 µL) were added to 96-well microplate, and l-ascorbic acid was the positive control. After incubation at 37 °C for 10 min, the absorbance was tested at 745 nm using a microplate reader. Scavenging rate was calculated according to Equation (1).

(1) $$\text{ABTS radical scavenging activity (\%) }=\frac{A_{C}\text{–}A_{S}}{A_{C}}\times 100$$

where A_C_ and A_S_ are the absorbance of the blank control and the compounds to be tested, respectively.

### 3.6. DPPH Radical Scavenging Activity

The scavenging ability on DPPH radical was conducted based on the method with some modifications [19]. The sample solution and DPPH methanol solution (20 µL:180 µL) were added to a 96-well microplate. l-ascorbic acid was the positive control. The absorbance at 517 nm was measured using a microplate reader after the solution had stood for 30 min at 37 °C under dark conditions. The calculation formula of DPPH radical scavenging activity is consistent with the formula of ABTS radical scavenging activity.

### 3.7. PTIO Radical Scavenging Activity

The PTIO radical scavenging activity was assayed by referring to relevant methods [52]. Briefly, PTIO radical solid (3 mg) was dissolved in 20 mL of methanol, and sample solution and PTIO methanol solution (40 µL:160 µL) were added to a 96-well microplate. The absorbance was determined at 585 nm using a microplate reader after 30 min of incubation and the scavenging rate was calculated on the basis of Equation (2).

(2) $$\text{PTIO radical scavenging activity (\%) }=[1-\frac{A_{S}}{A_{C}}]\times 100$$

where A_S_ is the absorbance of the compounds to be tested and A_C_ is the absorbance of the untreated control.

### 3.8. Tyrosinase Inhibitory Activity

The mushroom tyrosinase inhibitory activity was partially improved on the basis of reports [53,54]. Mushroom tyrosinase (400 U/mL) and l-tyrosine (3 mM) were added separately in 0.05 M potassium phosphate buffer (pH 6.5). A total of 80 µL of potassium phosphate buffer (pH 6.5), 80 µL of l-tyrosine (3 mM) solution, 20 µL of sample solution and 20 µL of mushroom tyrosinase (400 U/mL) were added to a 96-well microplate. The mixture reacted for 1 h at 37 °C. Kojic acid was selected as the positive control. The absorbance was measured at 490 nm using a microplate reader and Equation (3) was used to calculate the inhibition rate.

(3) $$\text{Tyrosinase inhibition activity (\%) }=\frac{A_{C}\text{–}A_{S}}{A_{C}}\times 100$$

Equation (3) is the absorbance of the test compound and A_C_ is the absorbance of the untreated control.

### 3.9. Acetylcholinesterase Inhibitory Activity

The experiment of acetylcholinesterase inhibition activity was slightly modified according to the literature [55]. Acetylcholinesterase (AChE) and acetylthiocholine iodide (ATCI) were dissolved in 0.1 M phosphate buffer (pH 8.0). 5,5′-Dithiobis-(2-nitrobenzoic acid) (DTNB) was prepared in 10 mL of 0.1 M phosphate buffer (pH 7.0) with a small amount of NaHCO_3_. A total of 120 µL of 0.1 M phosphate buffer (pH 8.0), 20 µL of 3 mM DTNB solution, 20 µL of sample solution and 20 µL of AChE (0.2 U/mL) were sequentially added to the 96-well microplate, and the mixture reacted for 10 min at 37 °C. The reaction was started by adding 20 µL of 3 mM ATCI and the mixture was incubated at 37 °C for 20 min. Donepezil was chosen as the positive control. The absorbance was tested at 412 nm and the inhibition rate was calculated based on Equation (4).

(4) $$\text{Acetylcholinesterase inhibition activity (\%) }=[1-\frac{\left(A_{S}\text{–}\mathrm{Aj}\right)}{A_{C}}]\times 100$$

where A_s_ and A_j_ are the absorbance of the compound to be tested and tested compound blanks, respectively, and A_C_ is the absorbance of the untreated control.

## 4. Conclusions

In conclusion, two new phenolic of ceroffesters A-B (**1**–**2**) and thirteen compounds (**3**–**15**) have been reported and isolated from *C. glabra* leaves. Their structures were identified mainly by NMR, UV, IR, HR-ESI-MS, ORD and ECD. Biologically, compounds **3**–**6**, **10** and **13** exhibited obvious antioxidant activities, and compounds **5**, **10** and **13** showed significant tyrosinase inhibitory activities. At an initial concentration of 1 mg/mL, compounds **3**, **5** and **6** demonstrated moderate inhibitory activities against acetylcholinesterase. These results indicate that *C. glabra *is a potent source of natural antioxidants that could be used in managing diseases involved with the overexpression of tyrosinase and acetylcholinesterase.

## Figures and Tables

**Table 1:** ABTS, DPPH and PTIO radical scavenging activities of **1**–**15**.

Compound	ABTS Radical Scavenging Activity (%) ^[a]^	IC_50_ (μM)	DPPH Radical Scavenging Activity (%) ^[a]^	IC_50_ (mM)	PTIO Radical Scavenging Activity (%) ^[a]^	IC_50_ (mM)
**1**	43.44 ± 0.75	>500	18.38 ± 1.16	>100	19.01 ± 0.78	>100
**2**	17.57 ± 0.32	>500	16.35 ± 0.99	>100	17.93 ± 0.44	>100
**3**	88.10 ± 0.10	94	59.08 ± 0.43	0.87	57.55 ± 0.62	2.56
**4**	86.14 ± 0.81	>500	39.46 ± 1.25	6.25	18.18 ± 0.99	44.27
**5**	88.19 ± 0.16	97	61.67 ± 0.46	0.59	37.43 ± 0.65	6.36
**6**	88.04 ± 0.43	108	60.32 ± 0.47	0.59	26.71 ± 0.61	14.74
**7**	31.43 ± 0.73	>500	17.70 ± 0.77	>100	11.17 ± 0.97	>100
**8**	68.42 ± 0.60	>500	37.77 ± 1.03	6.56	10.13 ± 0.77	>100
**9**	49.15 ± 0.56	>500	21.42 ± 1.43	>100	12.63 ± 1.03	27.64
**10**	88.20 ± 0.16	45	56.03 ± 0.79	0.56	67.73 ± 1.17	0.53
**11**	13.86 ± 0.57	>500	18.38 ± 1.06	>100	5.27 ± 0.56	>100
**12**	21.82 ± 0.10	>500	18.49 ± 2.86	34.87	21.31 ± 1.19	>100
**13**	88.19 ± 0.43	179	55.80 ± 0.44	0.77	27.07 ± 0.93	>100
**14**	15.67 ± 0.44	>500	9.47 ± 0.86	>100	21.44 ± 0.55	>100
**15**	67.06 ± 0.85	>500	16.23 ± 0.89	>100	21.17 ± 0.92	>100
l-ascorbic acid	88.24 ± 0.83	156	70.01 ± 0.34	0.44	70.65 ± 0.94	1.61

^[a]^ At initial concentration of 1 mg/mL. Results were expressed as means ± SEMs.

**Table 2:** Tyrosinase and acetylcholinesterase inhibitory activities of **1**–**15**.

Compound	Tyrosinase Inhibition (%) ^[a]^	IC_50_ (mM)	Compound	Acetylcholinesterase Inhibition (%) ^[a]^	IC_50_ (μM)
**1**	53.13 ± 0.65	2.63	**1**	38.43 ± 0.20	>1000
**2**	53.54 ± 0.90	2.59	**2**	39.46 ± 0.25	>1000
**3**	60.00 ± 1.49	1.39	**3**	85.27 ± 0.06	276
**4**	44.17 ± 0.85	4.69	**4**	68.13 ± 0.24	664
**5**	77.29 ± 1.52	0.64	**5**	83.65 ± 0.48	345
**6**	57.50 ± 0.82	1.69	**6**	82.21 ± 0.09	377
**7**	32.71 ± 1.51	9.24	**7**	41.69 ± 0.48	>1000
**8**	39.17 ± 0.86	4.96	**8**	40.11 ± 0.31	>1000
**9**	23.54 ± 1.04	NA	**9**	39.90 ± 0.33	>1000
**10**	66.04 ± 0.62	0.65	**10**	61.30 ± 0.38	374
**11**	29.79 ± 0.88	8.84	**11**	40.83 ± 0.41	>1000
**12**	35.63 ± 1.01	7.47	**12**	39.84 ± 0.53	>1000
**13**	80.00 ± 0.78	0.59	**13**	71.74 ± 0.40	>1000
**14**	37.71 ± 0.56	NA	**14**	47.18 ± 0.16	>1000
**15**	42.08 ± 0.54	4.04	**15**	39.91 ± 0.36	NA
kojic acid	84.38 ± 0.31	0.63	donepezil	91.17 ± 0.23	3.3

^[a]^ At initial concentration of 1 mg/mL. Results were expressed as means ± SEMs.

**Table 3:** ^1^H and ^13^C NMR data of compounds **1**, **2** and ceroffester D ^[a]^.

Position	1		2		Ceroffester D [34]	
	*δ*_C_	*δ*_H_ (mult, *J* in Hz)	*δ*_C_	*δ*_H_ (mult, *J* in Hz)	*δ*_C_	*δ*_H_ (mult, *J* in Hz)
1	127.1		127.0		127.0	
2	131.6	7.48 (d, 8.0)	131.4	7.46 (d, 8.4)	131.6	7.49 (d, 8.7)
3	117.0	6.82 (d, 8.0)	116.9	6.82 (d, 8.4)	117.0	6.85 (d, 8.7)
4	161.7		161.4		161.5	
5	117.0	6.82 (d, 8.0)	116.9	6.82 (d, 8.4)	117.0	6.85 (d, 8.7)
6	131.6	7.48 (d, 8.0)	131.4	7.46 (d, 8.4)	131.6	7.49 (d, 8.7)
7	148.3	7.73 (d, 16.0)	148.0	7.72 (d, 16.0)	148.2	7.73 (d, 15.9)
8	113.8	6.36 (d, 16.0)	113.9	6.36 (d, 16.0)	113.9	6.42 (d, 15.9)
9	167.8		167.9		167.9	
1′	169.3		170.4		169.1	
2′	74.8	5.57 (d, 2.4)	74.6	5.57 (d, 2.4)	75.3	5.62 (d, 2.9)
3′	72.0	4.81 (d, 2.4)	71.9	4.86 (d, 2.4)	72.1	4.77 (d, 2.9)
4′	172.4		172.6		172.2	
1′-OCH_3_	53.3	3.79 (s)			53.2	3.77 (s)
4′-OCH_3_	53.2	3.74 (s)	53.2	3.74(s)	53.2	3.82 (s)

^[a]^ 400 MHz for ^1^H and 100 MHz for ^13^C, recorded in CD_3_OD.

## Data Availability

Data are contained within the article and Supplementary Materials.

## Supplementary Materials

The following supporting information can be downloaded at: https://www.mdpi.com/article/10.3390/molecules27248667/s1, Figure S1: HR-ESI-MS spectrum of **1** (ceroffester A); Figure S2: UV spectrum of **1** (ceroffester A) in MeOH; Figure S3: IR spectrum of **1** (ceroffester A); Figure S4: ^1^H NMR spectrum (400 MHz) of **1** (ceroffester A) in CD_3_OD; Figure S5: ^13^C NMR spectrum (100 MHz) of **1** (ceroffester A) in CD_3_OD; Figure S6: DEPT 135 spectrum of **1** (ceroffester A) in CD_3_OD; Figure S7: HSQC spectrum of **1** (ceroffester A) in CD_3_OD; Figure S8: ^1^H-^1^H COSY spectrum of **1** (ceroffester A) in CD_3_OD; Figure S9: HMBC spectrum of **1** (ceroffester A) in CD_3_OD; Figure S10: NOESY spectrum of **1** (ceroffester A) in CD_3_OD; Figure S11: HR-ESI-MS spectrum of **2** (ceroffester B); Figure S12: UV spectrum of **2** (ceroffester B) in MeOH; Figure S13: IR spectrum of **2** (ceroffester B); Figure S14: ^1^H NMR spectrum (400 MHz) of **2** (ceroffester B) in CD_3_OD; Figure S15: ^13^C NMR spectrum (100 MHz) of **2** (ceroffester B) in CD_3_OD; Figure S16: DEPT 135 spectrum of **2** (ceroffester B) in CD_3_OD; Figure S17: HSQC spectrum of **2** (ceroffester B) in CD_3_OD; Figure S18: ^1^H-^1^H COSY spectrum of **2** (ceroffester B) in CD_3_OD; Figure S19: HMBC spectrum of **2** (ceroffester B) in CD_3_OD.
