# Supplementary material for: Natural Antioxidants, Tyrosinase and Acetylcholinesterase Inhibitors from Cercis glabra Leaves
Source: Molecules. 2022 Dec 7;27(24):8667. doi: 10.3390/molecules27248667 (PMC9782510; doi:10.3390/molecules27248667)
Supplement: Supplementary file 1 [file molecules-27-08667-s001.zip › molecules-2071082-supplementary.pdf]

Supporting Information

Natural Antioxidants, Tyrosinase and Acetylcholinesterase Inhibitors from *Cercis glabra* Leaves

Yueyue Lou [1,2,3,†], Ting Xu [1,2,3,†], Huaqiang Cao [1,2], Qiuyue Zhao [3], Pengpai Zhang [1,2,*] and Penghua Shu [3,*]

1 School of Life Sciences, Henan University, Kaifeng 475004, China

2 Engineering Research Center for Applied Microbiology of Henan Province, Kaifeng 475004, China

3 Food and Pharmacy College, Xuchang University, 88 Bayi Road, Xuchang 461000, China

* Correspondence: bio_apai@163.com (P.Z.); shupenghua@yeah.net (P.S.)

† These authors contributed equally to this work.

Figure S1. HR-ESI-MS spectrum of **1** (ceroffester A).

Figure S2. UV spectrum of **1** (ceroffester A) in MeOH.

Figure S3. IR spectrum of **1** (ceroffester A).

Figure S4. ^{1}H NMR spectrum (400 MHz) of **1** (ceroffester A) in CD_3OD.

Figure S5. ^{13}C NMR spectrum (100 MHz) of **1** (ceroffester A) in CD_3OD.

Figure S6. DEPT 135 spectrum of **1** (ceroffester A) in CD_3OD.

Figure S7. HSQC spectrum of **1** (ceroffester A) in CD_3OD.

Figure S8. ^{1}H-^{1}H COSY spectrum of **1** (ceroffester A) in CD_3OD.

Figure S9. HMBC spectrum of **1** (ceroffester A) in CD_3OD.

Figure S10. NOESY spectrum of **1** (ceroffester A) in CD_3OD.

Figure S11. HR-ESI-MS spectrum of **2** (ceroffester B).

Figure S12. UV spectrum of **2** (ceroffester B) in MeOH.

Figure S13. IR spectrum of **2**(ceroffester B).

Figure S14. ^{1}H NMR spectrum (400 MHz) of **2** (ceroffester B) in CD_3OD.

Figure S15. ^{13}C NMR spectrum (100 MHz) of **2** (ceroffester B) in CD_3OD.

Figure S16. DEPT 135 spectrum of **2** (ceroffester B) in CD_3OD.

Figure S17. HSQC spectrum of **2** (ceroffester B) in CD_3OD.

Figure S18. ^{1}H-^{1}H COSY spectrum of **2** (ceroffester B) in CD_3OD.

Figure S19. HMBC spectrum of **2** (ceroffester B) in CD_3OD.

Elemental Composition Report

Single Mass Analysis

Tolerance = 10.0 PPM / DBE: min = -1.5, max = 50.0
Element prediction: Off
Number of isotope peaks used for i-FIT = 3

Monoisotopic Mass, Even Electron Ions
8 formula(e) evaluated with 1 results within limits (up to 50 best isotopic matches for each mass)
Elements Used:
C: 15-15 H: 17-17 O: 0-10

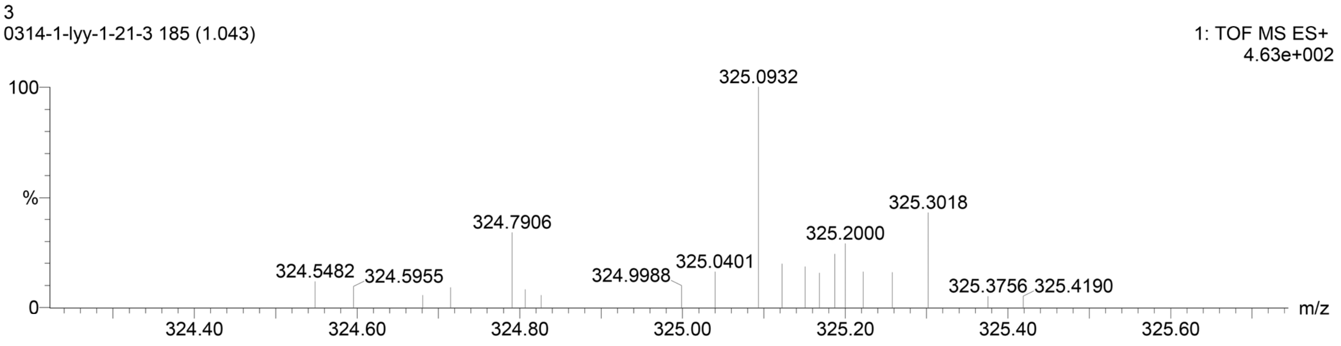

Minimum:				-1.5
Maximum:		5.0	10.0	50.0

Mass	Calc. Mass	mDa	PPM	DBE	i-FIT	Norm	Conf(%)	Formula
325.0932	325.0923	0.9	2.8	7.5	108.0	n/a	n/a	C15 H17 O8

Figure S1. HR-ESI-MS spectrum of **1** (ceroffester A).

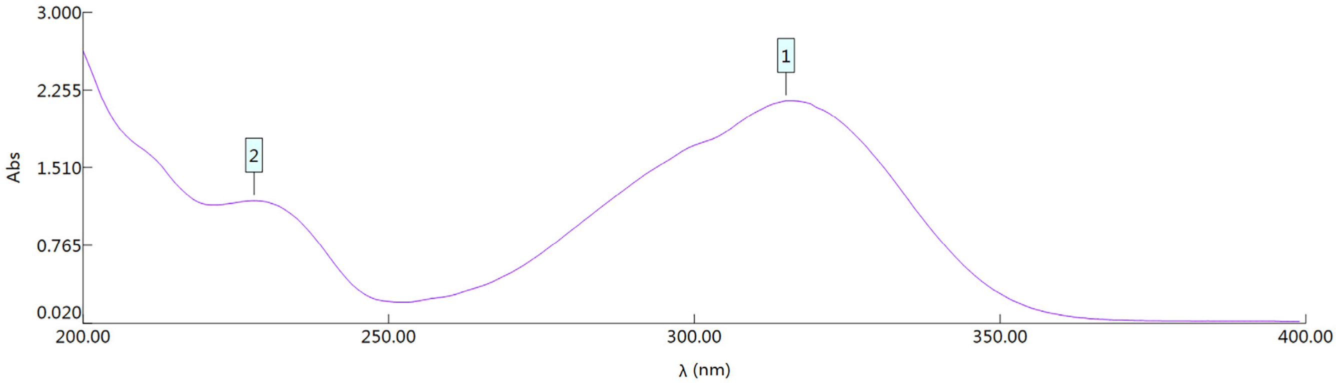

Peak	λ (nm)	Abs
1	315.00	2.154
2	228.00	1.196

Figure S2. UV spectrum of **1** (ceroffester A) in MeOH.

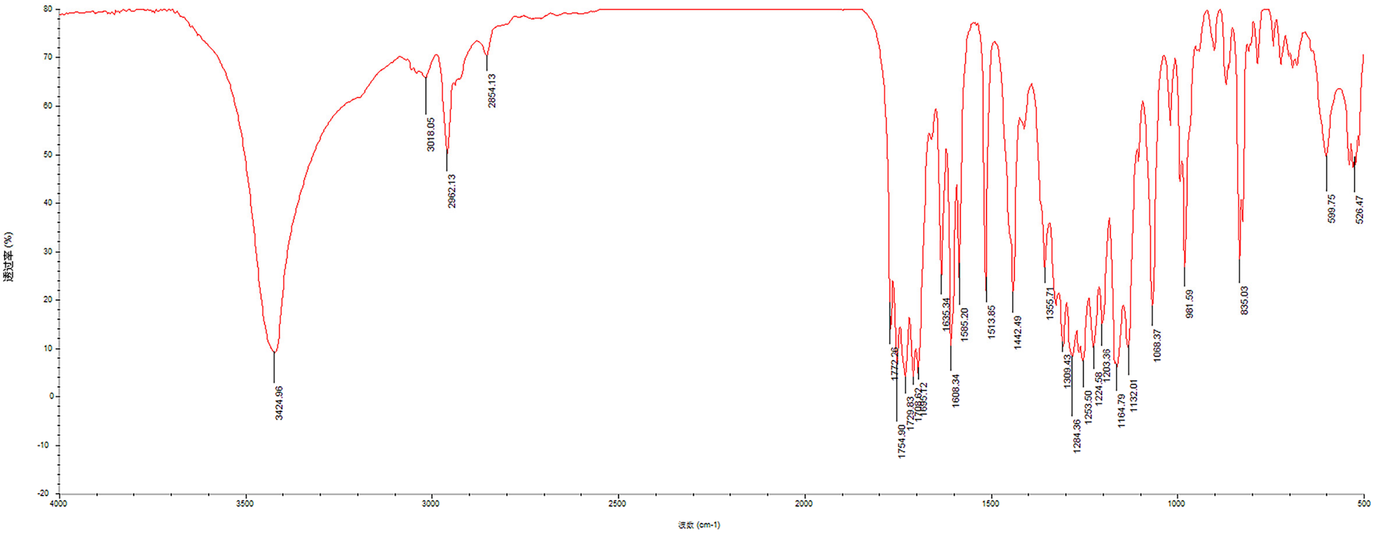

Figure S3. IR spectrum of **1** (ceroffester A).

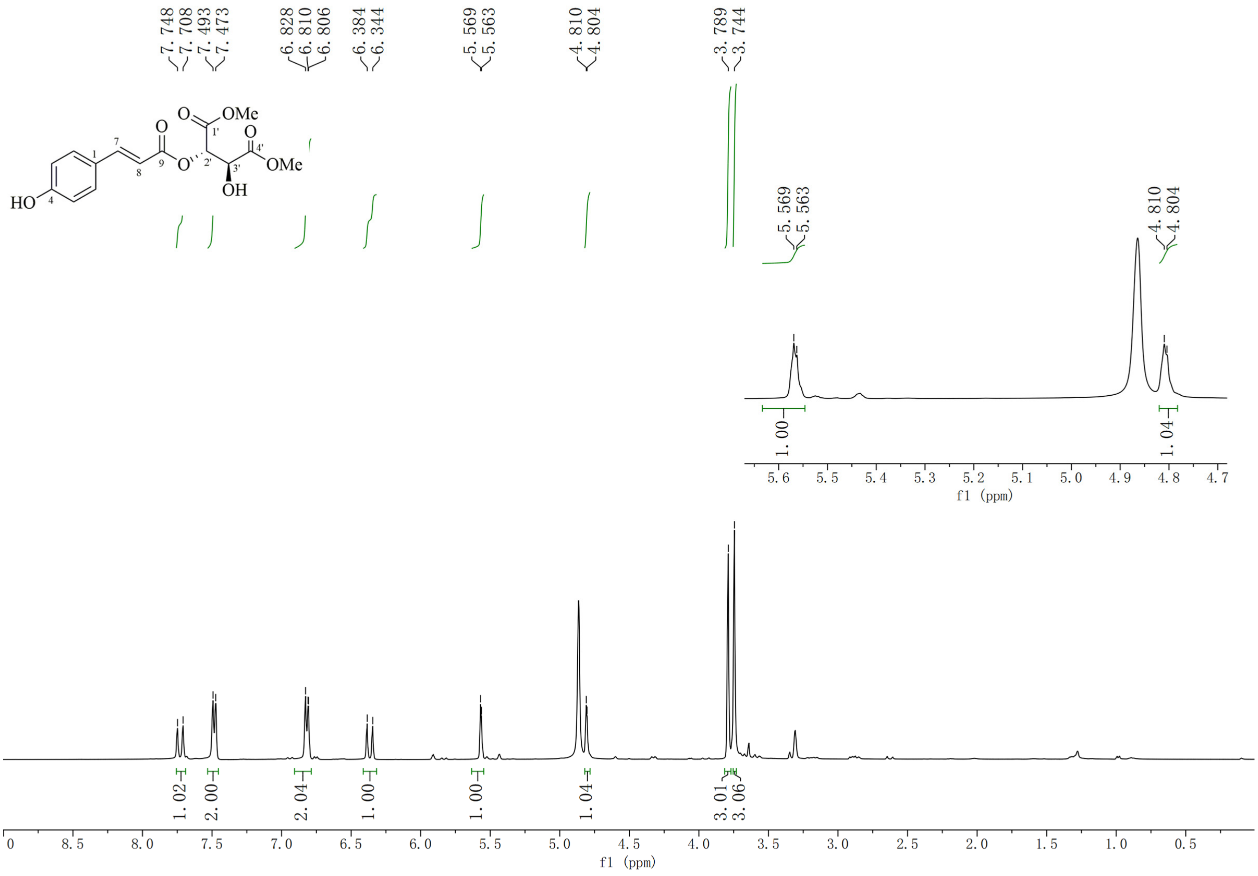

Figure S4. ^{1}H NMR spectrum (400 MHz) of **1** (ceroffester A) in CD_3OD.

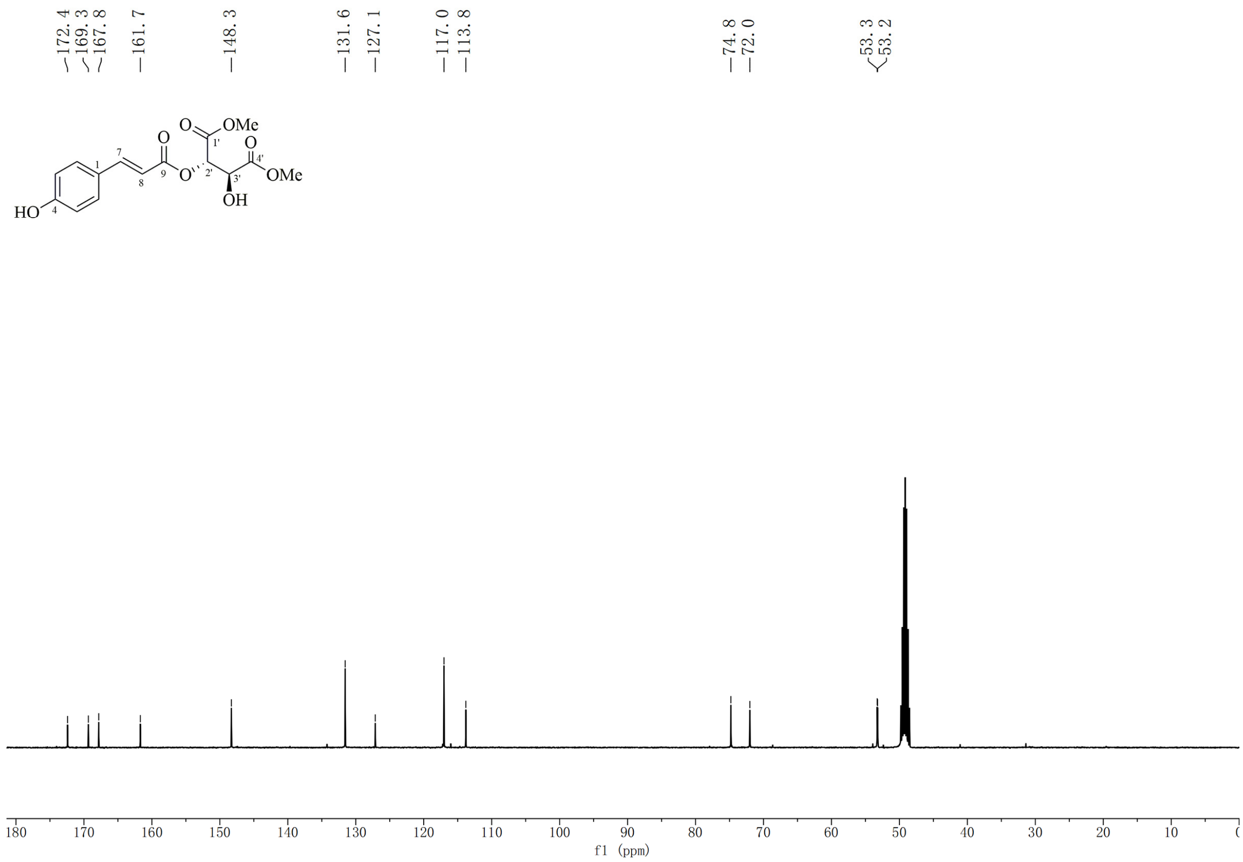

Figure S5. ^{13}C NMR spectrum (100 MHz) of **1** (ceroffester A) in CD_3OD.

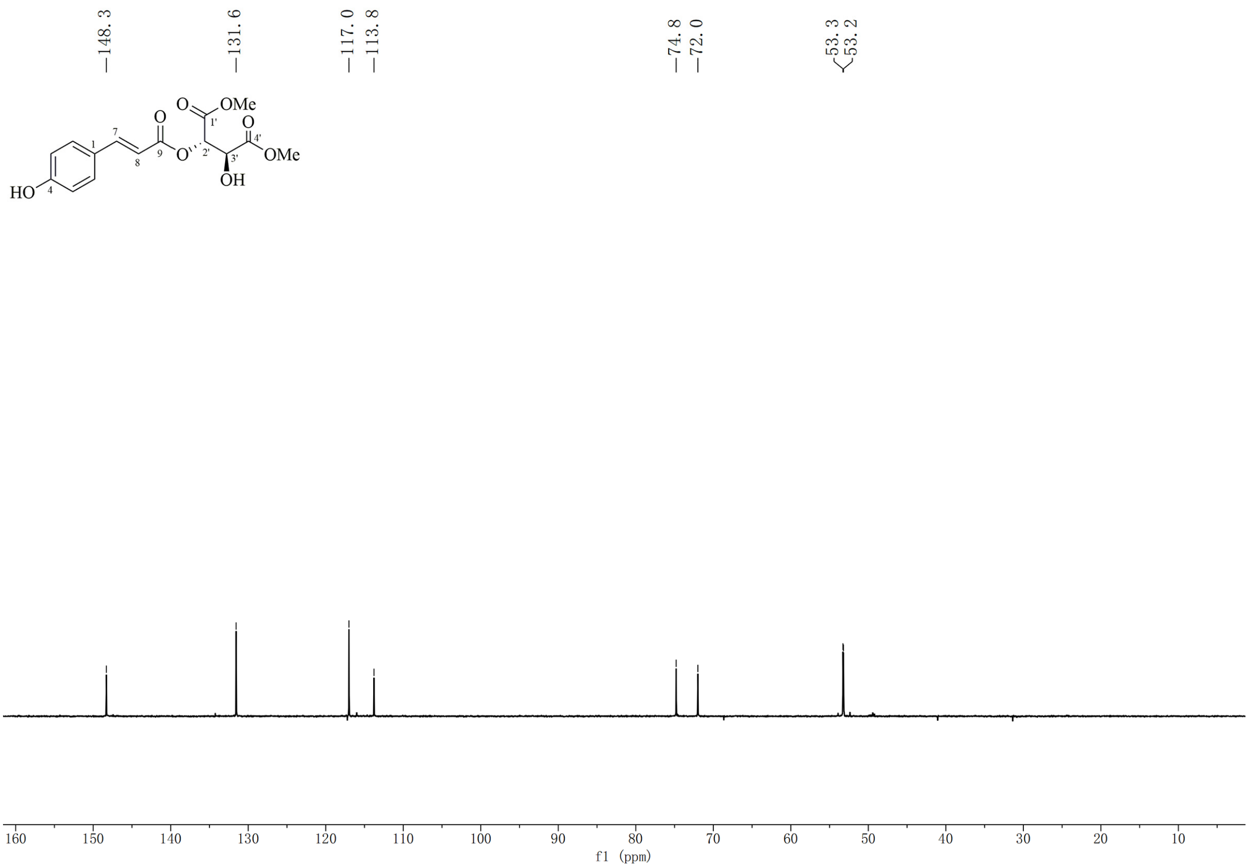

Figure S6. DEPT 135 spectrum of **1** (ceroffester A) in CD_3OD.

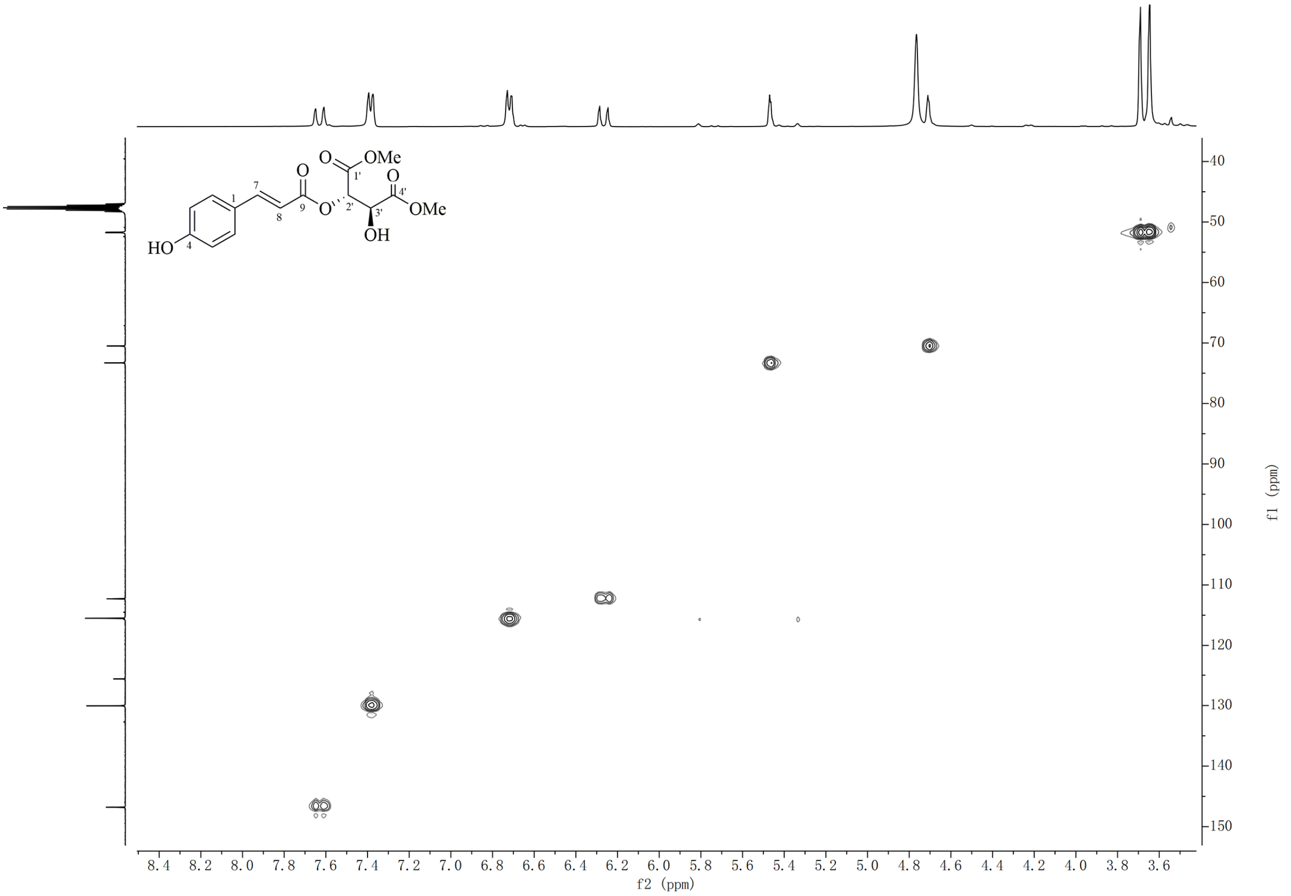

Figure S7. HSQC spectrum of **1** (ceroffester A) in CD_3OD.

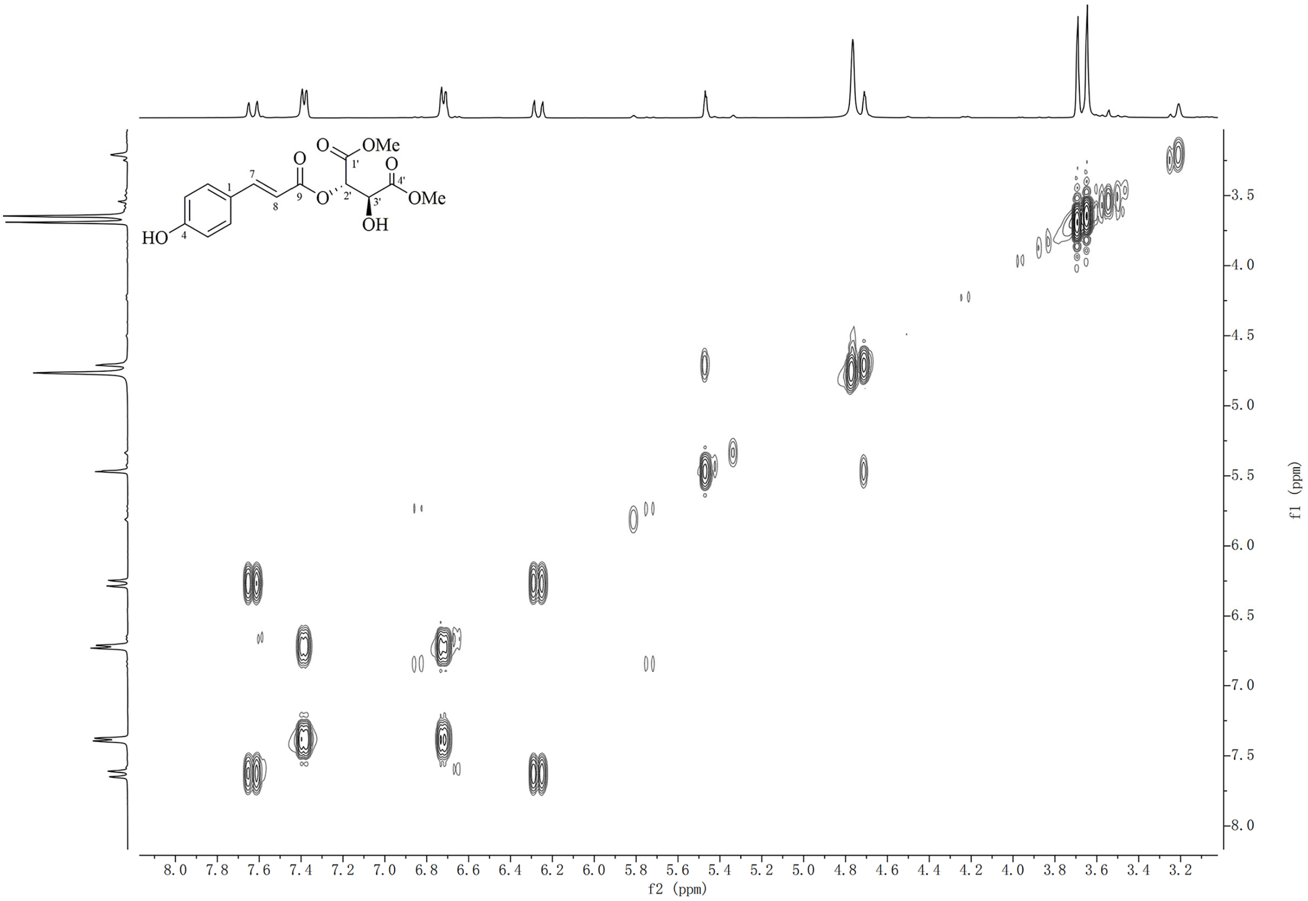

Figure S8. ^1H-^1H COSY spectrum of **1** (ceroffester A) in CD_3OD.

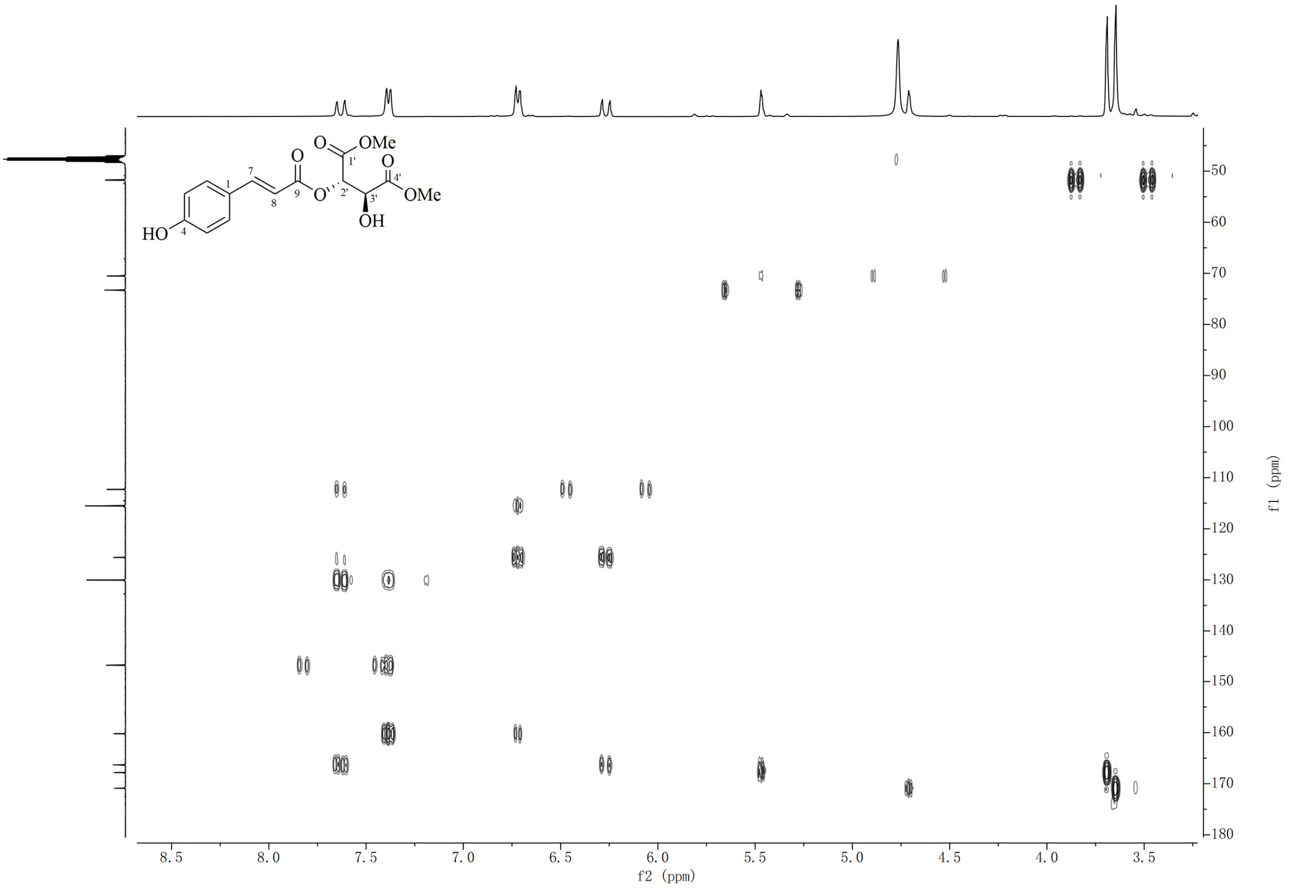

Figure S9. HMBC spectrum of **1** (ceroffester A) in CD_3OD.

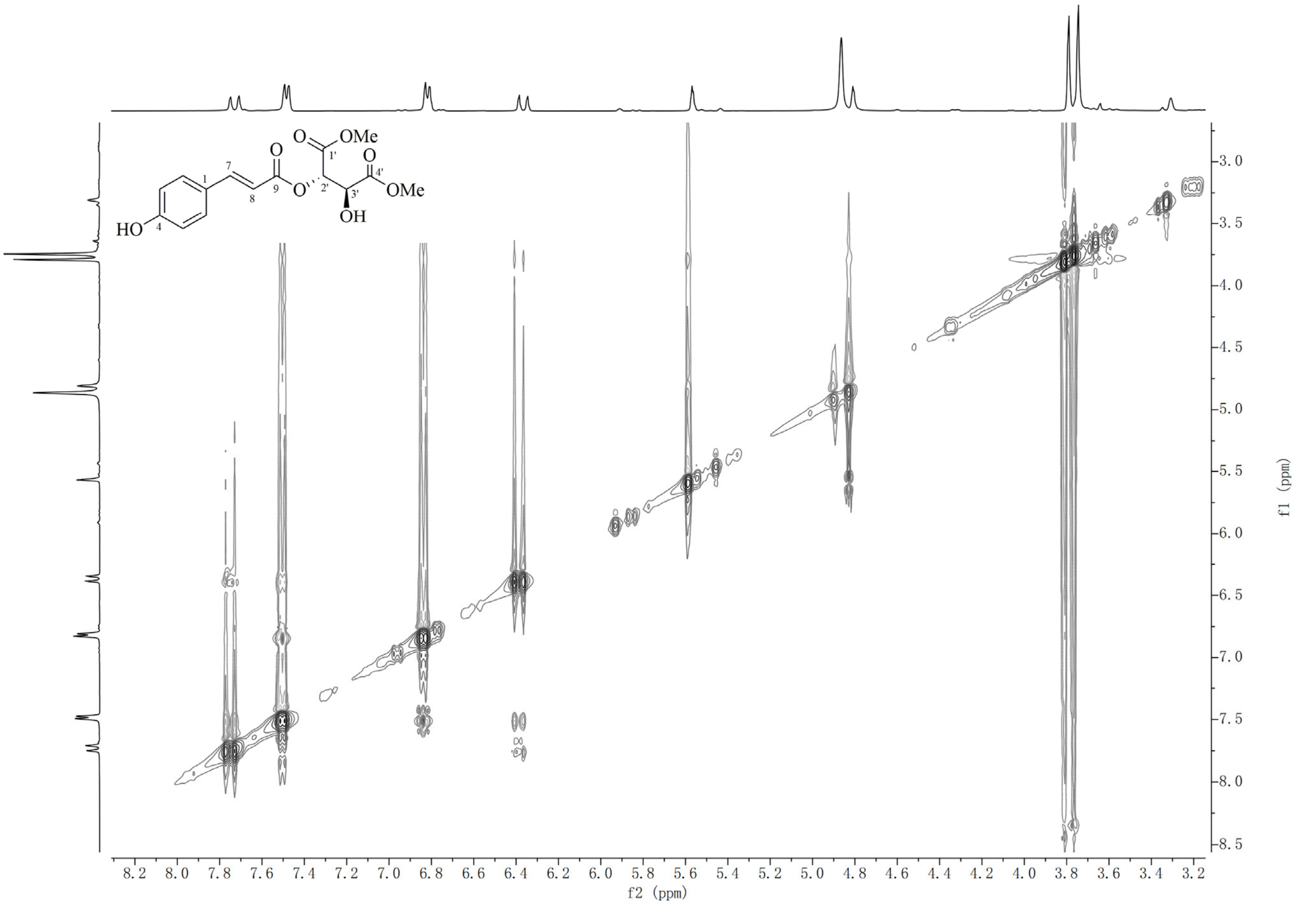

Figure S10. NOESY spectrum of **1** (ceroffester A) in CD_3OD.

Single Mass Analysis
Tolerance = 10.0 PPM / DBE: min = -1.5, max = 50.0
Element prediction: Off
Number of isotope peaks used for i-FIT = 3

Monoisotopic Mass, Even Electron Ions
8 formula(e) evaluated with 1 results within limits (up to 50 best isotopic matches for each mass)
Elements Used:
C: 14-14 H: 15-15 O: 0-10
3
0314-1-lyy-20-5 279 (1.560) 1: TOF MS ES+
2.06e+002

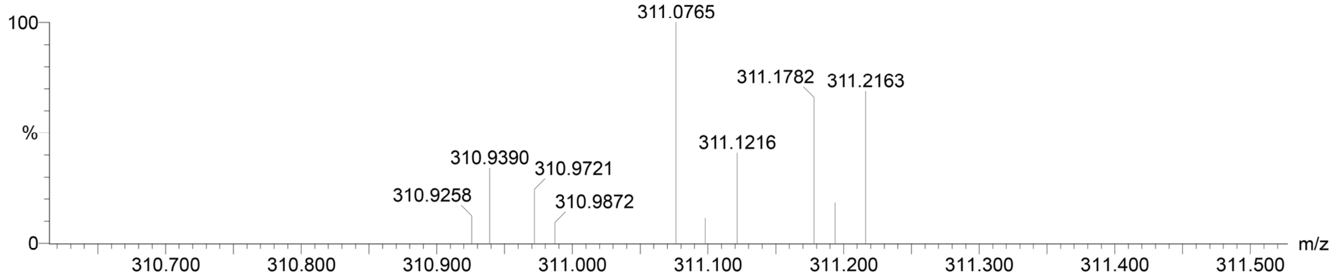

Minimum:				-1.5				
Maximum:		5.0	10.0	50.0				
Mass	Calc. Mass	mDa	PPM	DBE	i-FIT	Norm	Conf(%)	Formula
311.0765	311.0767	-0.2	-0.6	7.5	61.4	n/a	n/a	C14 H15 O8

Figure S11. HR-ESI-MS spectrum of **2** (ceroffester B).

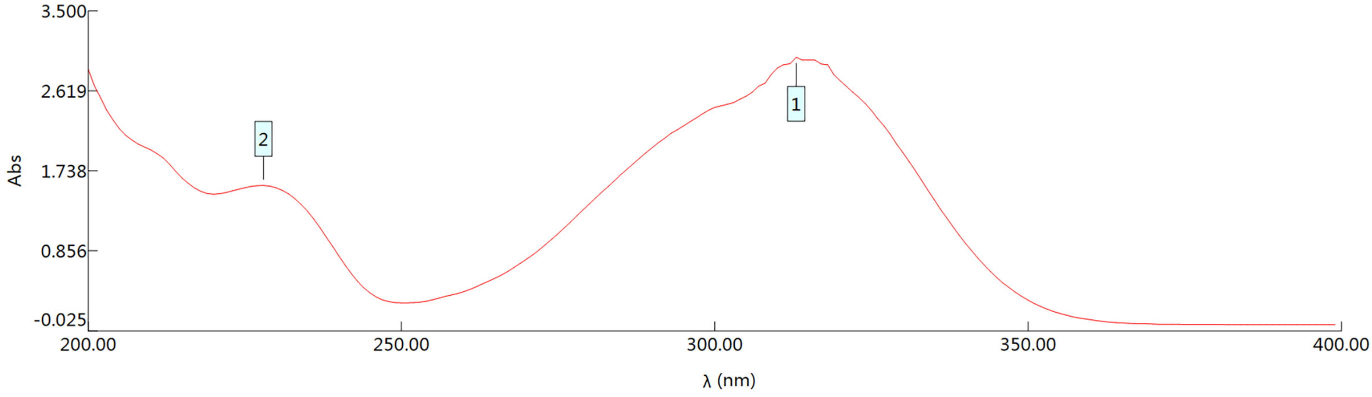

● Peak	λ (nm)	Abs
1	313.00	2.991
2	228.00	1.577

Figure S12. UV spectrum of **2** (ceroffester B) in MeOH.

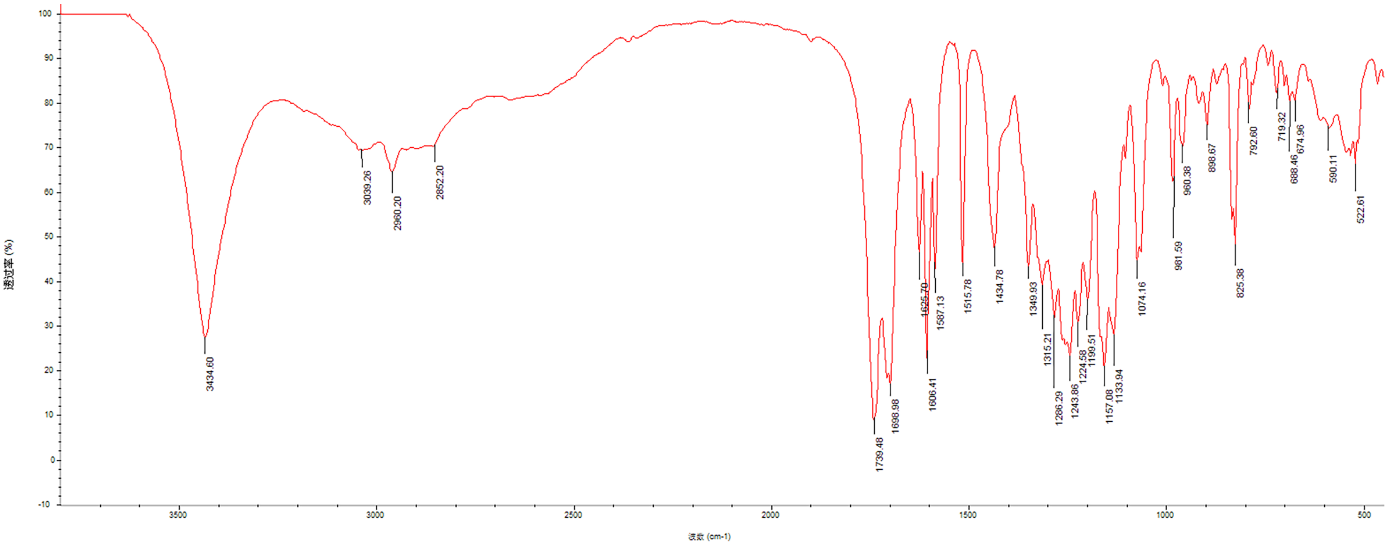

Figure S13. IR spectrum of **2** (ceroffester B).

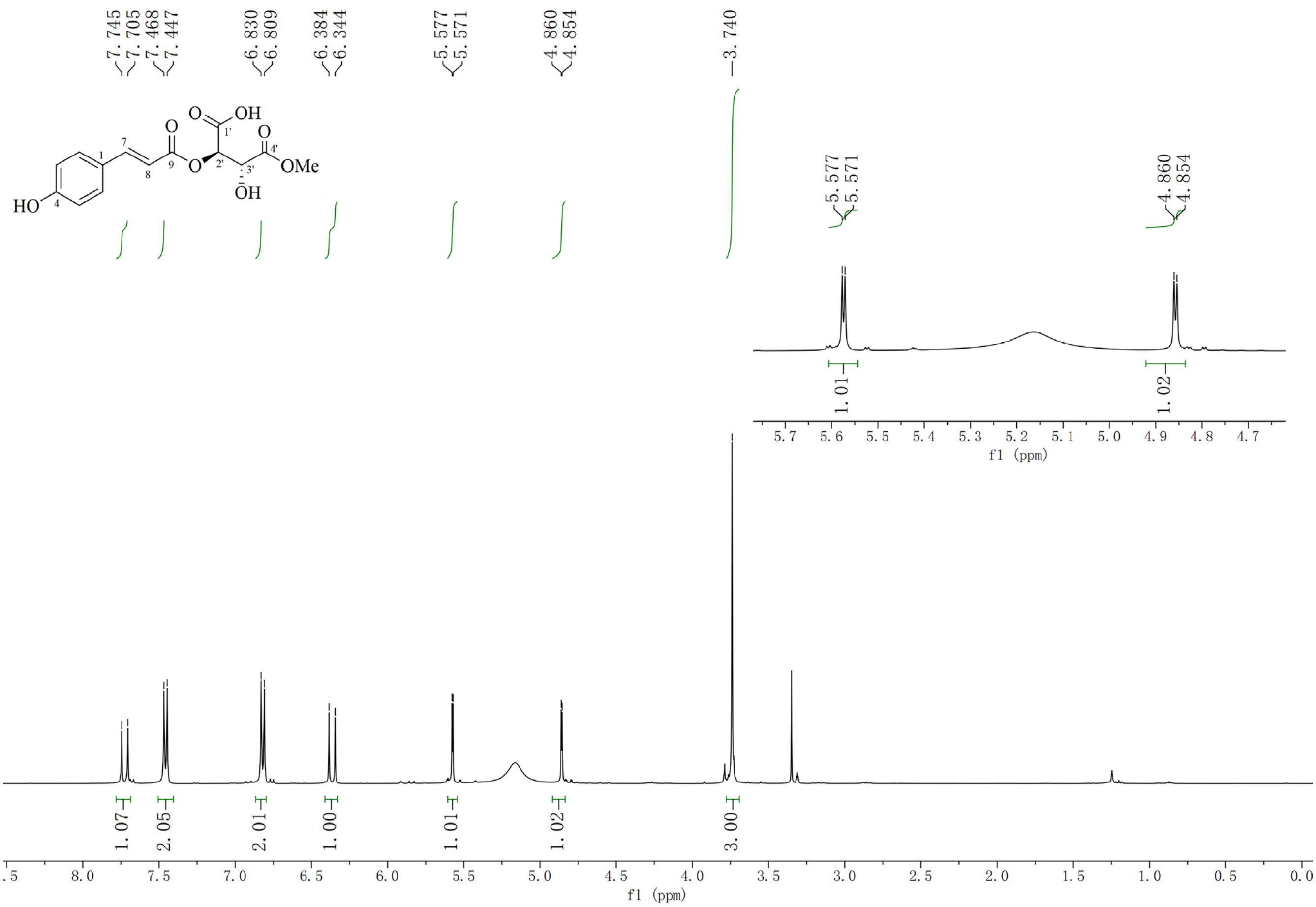

Figure S14. ^{1}H NMR spectrum (400 MHz) of **2** (ceroffester B) in CD_3OD.

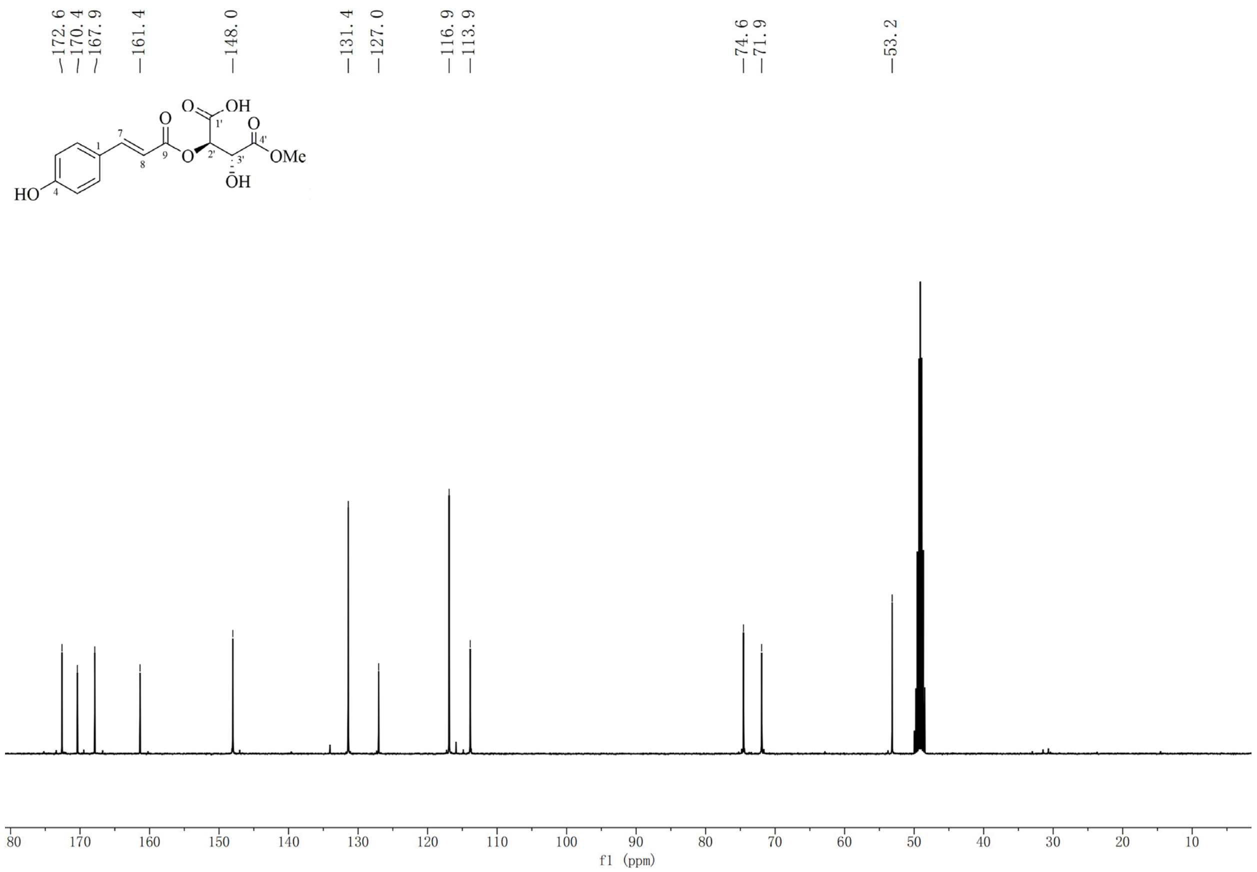

Figure S15. ^{13}C NMR spectrum (100 MHz) of **2** (ceroffester B) in CD_3OD.

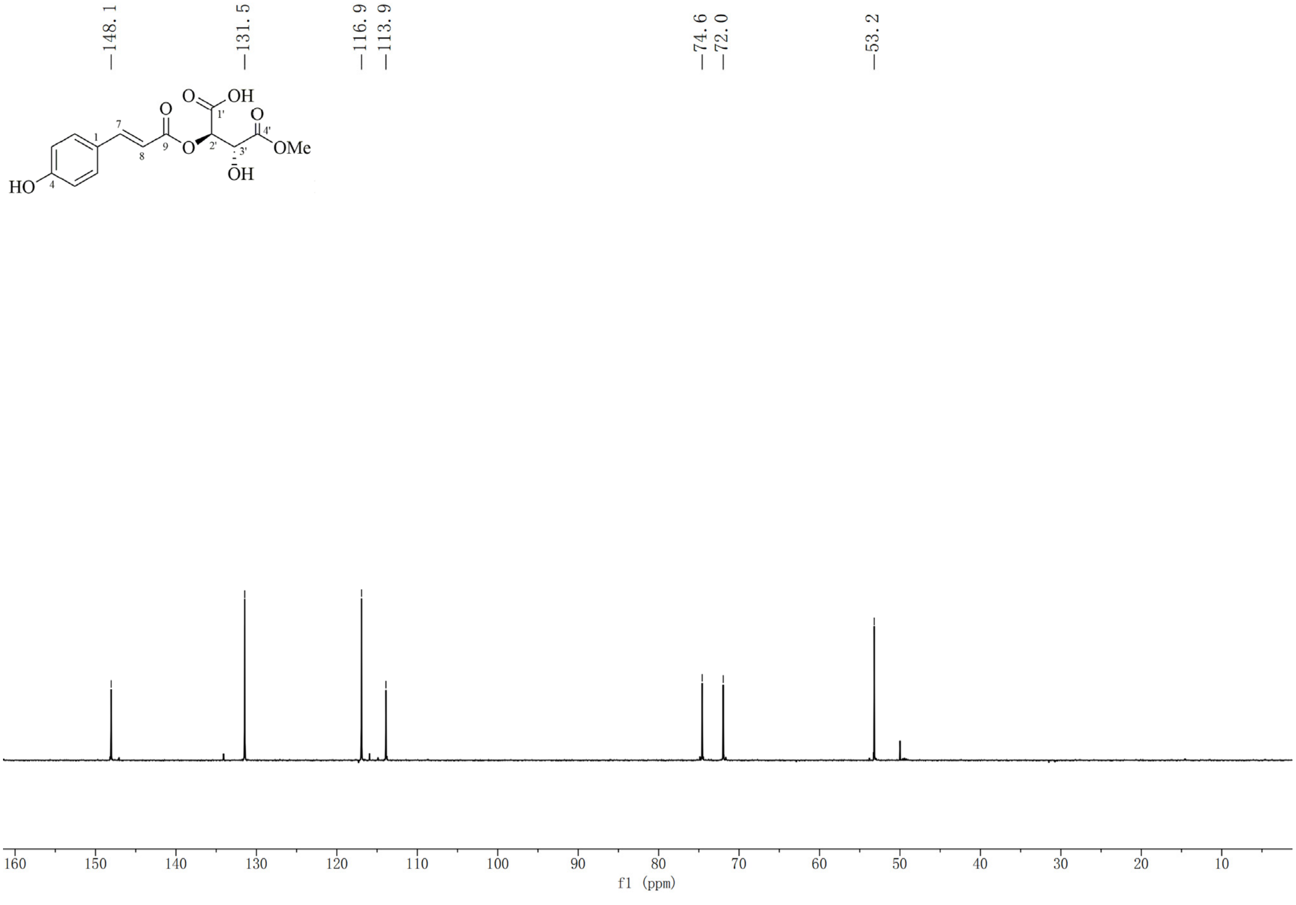

Figure S16. DEPT 135 spectrum of **2** (ceroffester B) in CD_3OD.

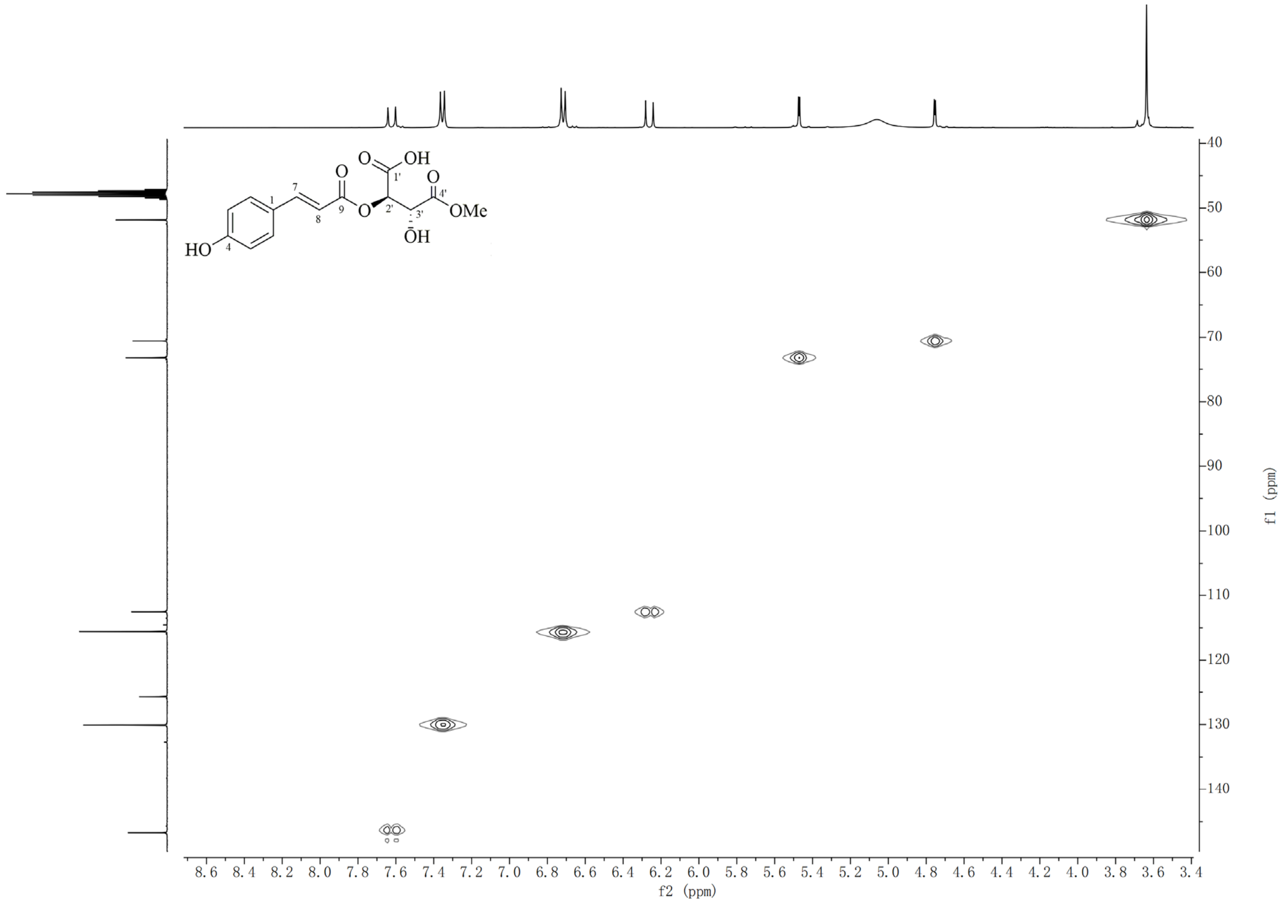

Figure S17. HSQC spectrum of **2** (ceroffester B) in CD_3OD.

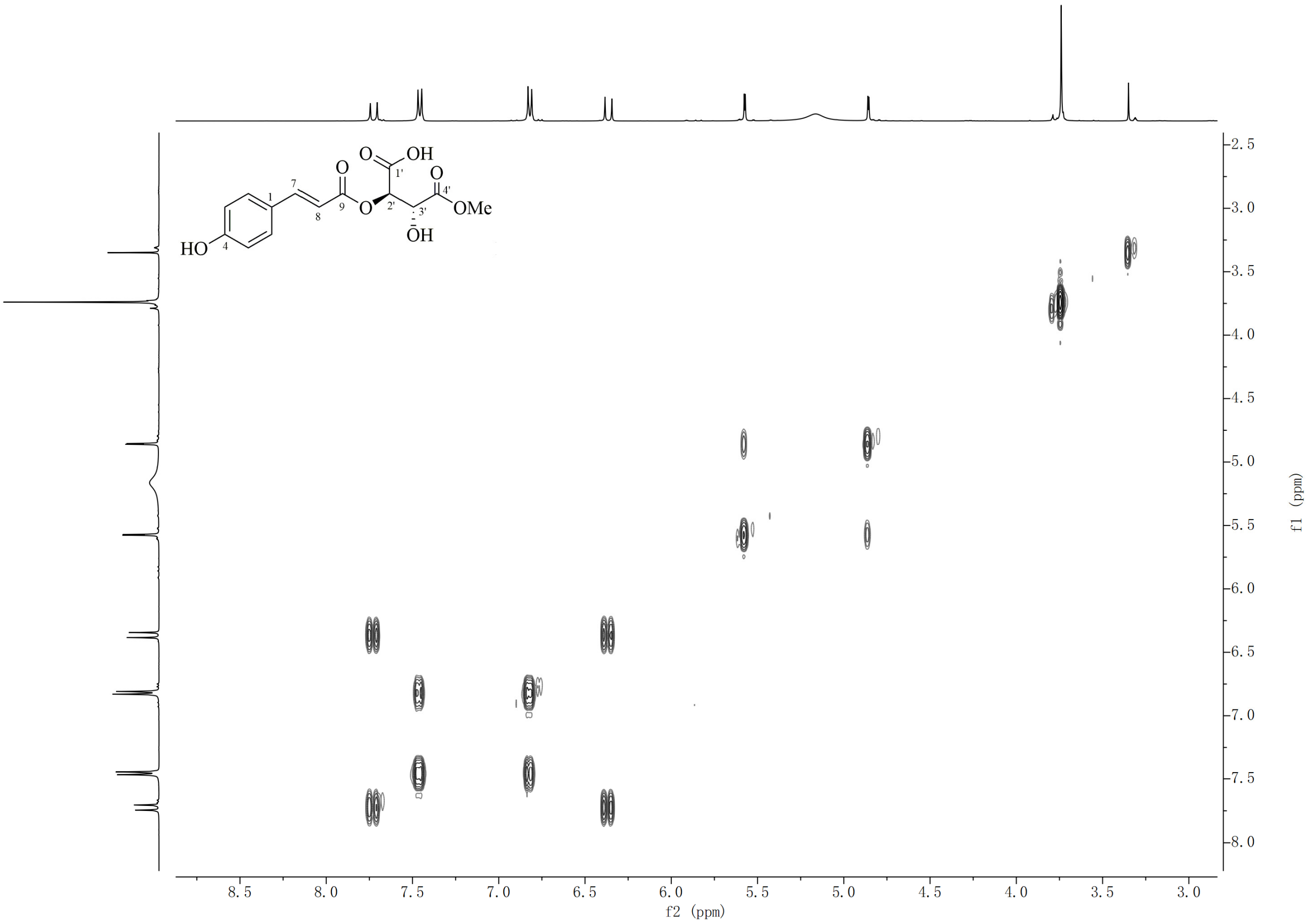

Figure S18. ^1H-^1H COSY spectrum of **2** (ceroffester B) in CD_3OD.

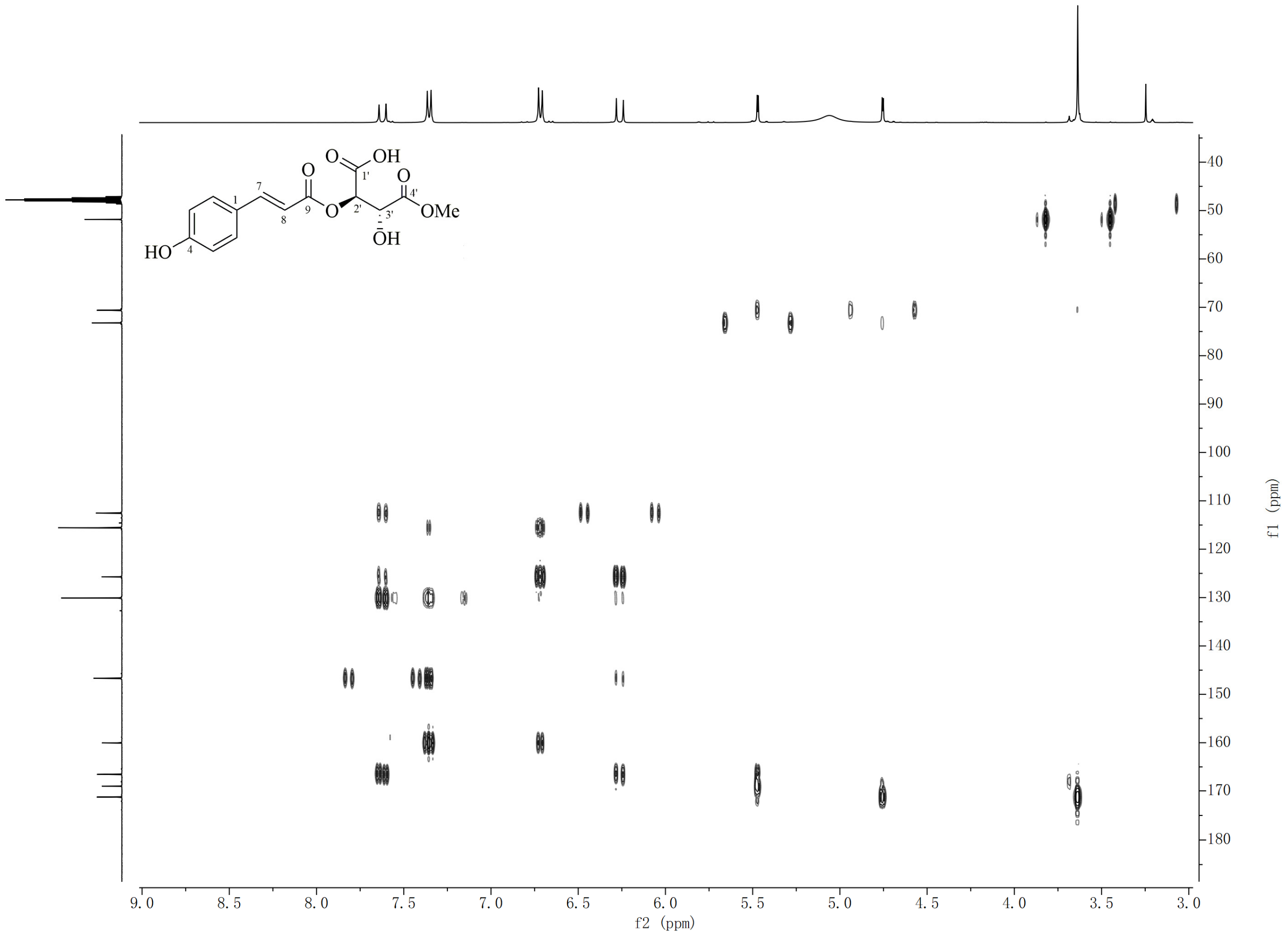

Figure S19. HMBC spectrum of **2** (ceroffester B) in CD_3OD.
